# Genome-wide gene expression tuning reveals diverse vulnerabilities of *M. tuberculosis*

**DOI:** 10.1016/j.cell.2021.06.033

**Published:** 2021-08-19

**Authors:** Barbara Bosch, Michael A. DeJesus, Nicholas C. Poulton, Wenzhu Zhang, Curtis A. Engelhart, Anisha Zaveri, Sophie Lavalette, Nadine Ruecker, Carolina Trujillo, Joshua B. Wallach, Shuqi Li, Sabine Ehrt, Brian T. Chait, Dirk Schnappinger, Jeremy M. Rock

**Affiliations:** 1 Laboratory of Host-Pathogen Biology, The Rockefeller University, New York, NY 10065, USA; 2 Laboratory of Mass Spectrometry and Gaseous Ion Chemistry, The Rockefeller University, New York, NY 10065, USA; 3 Department of Microbiology and Immunology, Weill Cornell Medicine, New York, NY 10065, USA

**Keywords:** Mycobacterium tuberculosis, Mycobacterium smegmatis, Essential genes, Vulnerability, Bayes Theorem, Mass Spectrometry, CRISPR-Cas Systems, Drug Development

## Abstract

Antibacterial agents target the products of essential genes but rarely achieve complete target inhibition. Thus, the all-or-none definition of essentiality afforded by traditional genetic approaches fails to discern the most attractive bacterial targets: those whose incomplete inhibition results in major fitness costs. In contrast, gene “vulnerability” is a continuous, quantifiable trait that relates the magnitude of gene inhibition to the effect on bacterial fitness. We developed a CRISPR interference-based functional genomics method to systematically titrate gene expression in *Mycobacterium tuberculosis* (Mtb) and monitor fitness outcomes. We identified highly vulnerable genes in various processes, including novel targets unexplored for drug discovery. Equally important, we identified invulnerable essential genes, potentially explaining failed drug discovery efforts. Comparison of vulnerability between the reference and a hypervirulent Mtb isolate revealed incomplete conservation of vulnerability and that differential vulnerability can predict differential antibacterial susceptibility. Our results quantitatively redefine essential bacterial processes and identify high-value targets for drug development.

## Highlights

• Titratable CRISPRi enables quantification of target vulnerability in mycobacteria
• Essential genes and processes vary widely in their vulnerability
• Differential vulnerability predicts differential antibacterial susceptibility
• Generalizable approach allows prioritization of high-value targets for drug discovery

## Graphical abstract

**Figure:**
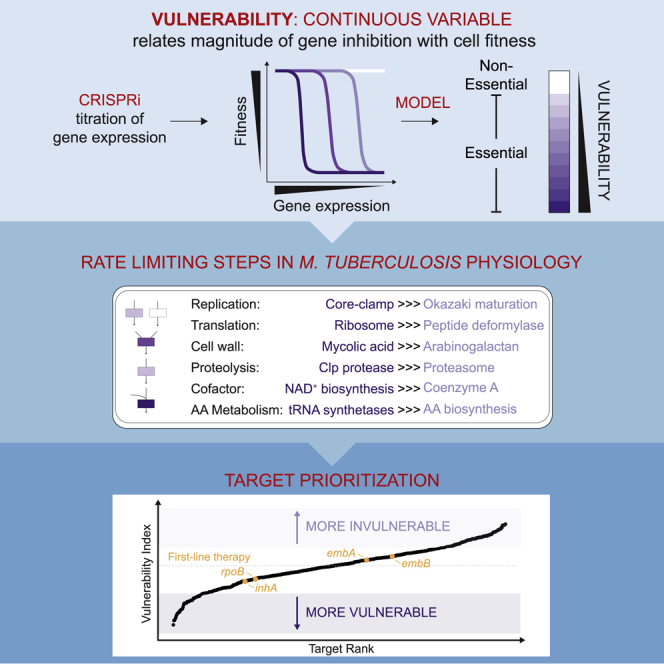

## Teaser

Conventional genetic approaches rely on complete gene inactivation to identify potential antimicrobial targets. In contrast to this all-or-nothing effect, small molecules rarely achieve complete target inhibition. Here, a CRISPR interference system using *M. tuberculosis* as a model organism is used to titrate gene expression and uncover gene vulnerability, redefining the concept of essential genes and identifying antimicrobial targets.

## Introduction

Essential bacterial genes orchestrate core biological processes and represent the targets of nearly all antibacterial drugs. Transposon insertion sequencing (TnSeq) and gene deletion typically treat gene essentiality as a binary variable: a gene is either essential for fitness in a given condition or it is non-essential. However, there is growing appreciation that partial inhibition of some essential genes results in strong fitness costs, whereas other essential genes can tolerate substantial inhibition with little effect on bacterial fitness (Hawkins et al., 2020; Jost et al., 2020; Keren et al., 2016; Wei et al., 2011). This expression-fitness relationship is defined as gene vulnerability (Barry et al., 2009; Wei et al., 2011). Vulnerability relates the magnitude of gene expression inhibition with the resulting decrease in organismal fitness, thus describing gene essentiality as a continuous trait. Despite the growing appreciation of variable expression-fitness relationships, quantification of gene vulnerability remains intractable with traditional genetic approaches and has yet to be defined systematically for any pathogen.

Understanding vulnerability is important for the study and targeting of the global pathogen *Mycobacterium tuberculosis* (Mtb). Mtb, the etiological agent of tuberculosis (TB), is one of the leading causes of death because of infectious disease and accounts for one-third of all deaths associated with antimicrobial resistance (WHO, 2020). Quantification of gene vulnerability would advance our understanding of Mtb by identifying the rate-limiting steps in Mtb physiology. This information, in turn, would enable prioritization of highly vulnerable genes and de-prioritization of highly invulnerable genes for antibacterial discovery. Although target-based drug discovery has yielded anti-infective agents undergoing clinical trials (Jarvest et al., 2002; Llanos-Cuentas et al., 2018; Payne et al., 2002), this drug discovery modality has been largely disappointing for antibacterial agents (Payne et al., 2007), and increasing its success rate would be impactful.

To enable quantification of gene vulnerability in Mtb, we developed a genome-scale *S. thermophilus* Cas9 (Sth1dCas9)-based CRISPR interference (CRISPRi) platform capable of systematically tuning endogenous gene expression levels over two orders of magnitude and monitoring the resulting bacterial fitness. We developed a mathematical framework to describe bacterial fitness as a function of predicted inhibition of target gene expression. Applying these approaches, we quantified vulnerability for nearly all essential genes in two Mtb strains and the model bacterium *M. smegmatis* (Msmeg) and used these results to define rate-limiting steps in mycobacterial physiology, analyze conservation of vulnerable and invulnerable Mtb gene sets, and identify promising and unexplored potential drug targets. This work provides a technical and conceptual framework for genome-scale assessment of gene vulnerability in diverse bacterial pathogens and a roadmap for prioritization of targets for drug discovery.

## Results

### Development and validation of genome-scale CRISPRi in Mtb

**Figure 1:**
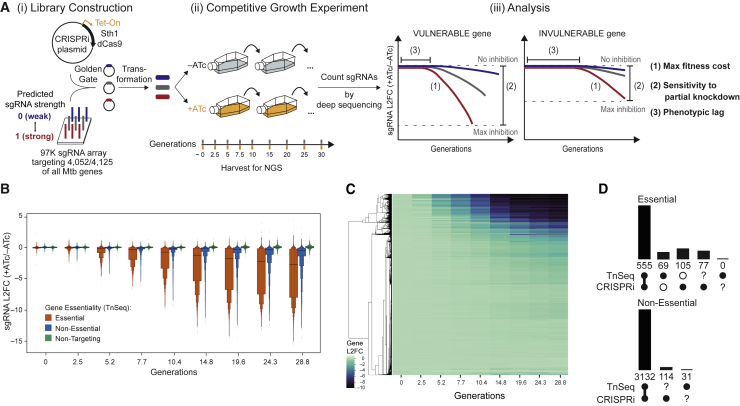
Genome-scale CRISPRi fitness profiling in Mtb (A) Experimental design to quantify Mtb gene vulnerability. (i) The Mtb CRISPRi library was built by cloning an sgRNA oligo array into an anhydrotetracycline (ATc)-inducible Sth1dCas9 vector. The library was designed to target all possible Mtb genes with sgRNAs of varying predicted knockdown efficiencies. (ii) Cultures were passaged for approximately 30 generations in the presence (CRISPRi on) or absence of ATc. At the indicated time points, genomic DNA was harvested and sgRNA targeting sequences amplified for next-generation sequencing. (iii) The relative fitness of individual strains was quantified by the sgRNA log2 fold change (L2FC) over time (+ATc/–ATc). Relative fitness values were then used to quantify three parameters that define target vulnerability: (1) maximum fitness cost, (2) sensitivity to partial knockdown, and (3) the phenotypic lag between the timing of CRISPRi induction and onset of a fitness defect. (B) Boxen plots (mean and quantiles) comparing time-dependent changes in L2FC values of sgRNAs targeting genes defined as Essential (n = 63,867) or Non-Essential (n = 29,609) by TnSeq and of control Non-Targeting sgRNAs (n = 1,658). sgRNAs targeting TnSeq Uncertain genes (n = 563) are not shown. (C) Hierarchical clustering of gene level depletion from the experiment described in (A). Each row represents a single targeted Mtb gene. (D) Bar chart showing the overlap between gene calls by TnSeq and CRISPRi. 42 genes in the Mtb genome cannot be called by either method. See also Figure S1 and Table S1.

**Figure S1:**
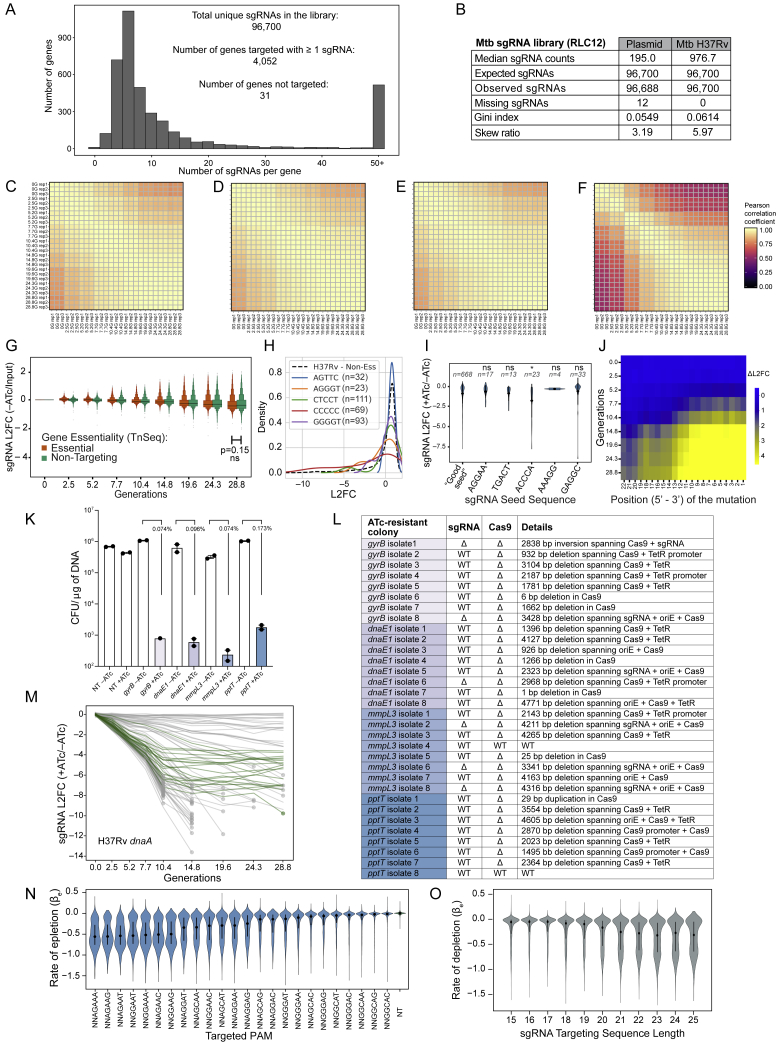
Genome-scale CRISPRi fitness profiling in Mtb H37Rv, related to Figures 1 and 2, STAR Methods and Table S1 (A) Histogram depicting the number of sgRNAs per gene in the Mtb CRISPRi library (RLC12; Addgene #163954). (B) Next generation sequencing quality-control metrics for the Mtb CRISPRi library. The “Plasmid” column depicts metrics for the RLC12 plasmid library following cloning and isolation from *E. coli*. The “H37Rv Mtb” column depicts library metrics following transformation and expansion in Mtb H37Rv. Skew ratio represents the ratio between top and bottom 10% of sgRNA counts. (C-F) Correlation heatmap of the triplicate screens depicted in Figure 1A. Panel (C) depicts the correlation between non-targeting sgRNAs in the –ATc cultures; panel (D) depicts the correlation between non-targeting sgRNAs in the +ATc cultures; panel (E) depicts the correlation between TnSeq essential gene targeting sgRNAs in the –ATc cultures; panel (F) depicts the correlation between TnSeq essential gene targeting sgRNAs in the +ATc cultures. G, generation. (G) Boxen plots comparing time-dependent changes in sgRNA L2FC values (mean ± quantiles) comparing –ATc to Input (i.e., generation 0). sgRNAs are grouped according to whether they target genes defined as Essential by TnSeq (n = 63,867 sgRNAs) or Non-Targeting sgRNAs (n = 1,658). ns, not significant. (H) Density plot to detect potential new “bad-seed” sequences. The plot shows the L2FC (+ATc/–ATc at generation 24.3) of all sgRNAs targeting non-essential genes (dashed line), and sgRNAs targeting non-essential genes that contain the indicated sgRNA seed sequences (defined as the five PAM-proximal nucleotides of the sgRNA targeting sequence) displaying the strongest depletion from the library. See STAR Methods “Estimate of the “bad-seed” effect” for more detail. (I) Violin plot showing the behavior of sgRNAs containing the strongest “bad-seed” sequences identified for SpydCas9 (Cui et al., 2018). Only sgRNAs targeting a CRISPRi non-essential gene were analyzed. sgRNAs with a PAM-proximal ‘ACCCA’ sequence (n = 24) show some evidence for target-independent depletion (i.e., "bad-seed" behavior). Dot and error bars represent mean and SD. ^∗^p = 0.021; ns, not significant. (J) Heatmap showing the behavior of mismatched sgRNAs in the competitive fitness experiment depicted in Figure 1A. ΔL2FC represents the difference in depletion between essential gene-targeting sgRNAs with perfectly matching targeting sequences and the corresponding mismatched sgRNAs. Mismatched sgRNAs contain mismatches between the sgRNA targeting sequence and the gene target at the indicated position (x axis; 22 is the sgRNA nucleotide furthest from the PAM). Mismatched sgRNAs were not designed but were the result of errors during library synthesis or cloning. (K) Frequency of ATc-resistant colonies that occur after transformation of four unique sgRNAs targeting the essential genes *gyrB* (*ms0005)*, *dnaE1* (*ms3178)*, *mmpL3* (*ms0250),* and *pptT* (*ms2648)* in Msmeg. Dots represent transformations performed in biological duplicate; error bars indicate median ± 95% CI. CFU, colony forming unit; NT, non-targeting. (L) Table summarizing the mutations observed in the CRISPRi plasmid in independent ATc-resistant colonies. All but two isolates show unique deletions, duplications, or an inversion (all generically marked as Δ to indicate lack of CRISPRi functionality) within the sgRNA, Cas9, or both. WT, wild-type; TetR, Tet repressor protein; oriE, *E. coli* origin of replication. (M) Line plot showing all sgRNAs targeting *dnaA* (*rv0001*) in the Mtb H37Rv CRISPRi fitness experiment. “Flatliner” sgRNAs of presumed CRISPRi-resistant subpopulations are indicated in green. See STAR Methods for details. (N and O) Distribution of sgRNA depletion slopes (β_e_) for sgRNAs targeting essential genes (n = 63,867 sgRNAs) stratified by targeted PAM sequence (N) or sgRNA targeting sequence length (O). Black dots and lines show the median and 25%–75% percentiles. Dot and error bars represent mean and SD. NT, non-targeting.

Quantification of target vulnerability requires predictable and titratable reduction of gene expression and determination of the resulting effect on bacterial fitness (Figure 1A). We reasoned that recently developed CRISPRi methods (Choudhary et al., 2015; Rock et al., 2017; Singh et al., 2016) could meet these demands. To comprehensively define target vulnerability, we first applied our Sth1dCas9 CRISPRi platform (Rock et al., 2017) to develop genome-scale CRISPRi in Mtb. We constructed an Mtb CRISPRi library designed to target all annotated Mtb genes with single guide RNAs (sgRNAs) of varying predicted knockdown efficiencies (Figure 1Ai). Knockdown tuning was achieved in two ways. First, we used the ability of Sth1dCas9 to recognize non-canonical protospacer adjacent motifs (PAMs) that lead to a gradient of target knockdown (Rock et al., 2017). Second, we varied the length of the sgRNA targeting sequence to modulate the extent of complementarity between the sgRNA and DNA target, further influencing target knockdown efficiency (Qi et al., 2013). The CRISPRi system is transcriptionally induced by anhydrotetracycline (ATc). This CRISPRi library is biased toward sgRNAs targeting predicted *in vitro* essential genes (DeJesus et al., 2017) because knockdown of these genes is predicted to reduce bacterial fitness and enable vulnerability quantification. The final library consists of 96,700 unique sgRNAs targeting 98.2% of all annotated Mtb genes and 1,658 non-targeting control sgRNAs (Figures S1A and S1B).

After cloning and transformation of the CRISPRi library into the Mtb strain H37Rv, triplicate cultures were passaged for approximately 30 generations in the presence or absence of ATc (Figure 1Aii). Every 2.5 or 5 generations, we harvested genomic DNA, analyzed sgRNA abundance by deep sequencing, and calculated the log2 fold change (L2FC) of sgRNA read counts ± ATc. Growth phenotypes were well correlated among triplicate screens (Figures S1C–S1F). Consistent with TnSeq predictions (DeJesus et al., 2017), sgRNAs targeting essential genes exhibited greater depletion, on average, than sgRNAs targeting non-essential genes and non-targeting controls (Figure 1B). We found no evidence of fitness defects in the absence of ATc, demonstrating the tight regulation of our ATc-inducible system (Figure S1G), and minimal evidence of fitness defects from bad sgRNA seeds, a poorly understood sequence-specific toxicity determined by the five PAM-proximal bases of the sgRNA (Cui et al., 2018; Figures S1H and S1I). Sth1dCas9 CRISPRi was specific, as demonstrated by the 4-log increase in fitness comparing sgRNAs with mismatches in the 10 PAM-proximal nucleotides with their perfectly matched counterparts (Figure S1J).

Hierarchical clustering of gene-level L2FC over time showed strong depletion for a subset of Mtb genes, consistent with the expected behavior for essential genes (Figure 1C). To benchmark genome-scale CRISPRi against TnSeq (DeJesus et al., 2017), we used a modified resampling approach to define CRISPRi essential genes. 89% of TnSeq essential calls (555 of 624) and 98% of non-essential calls (3,132 of 3,196) are shared with CRISPRi (Figure 1D). Of the CRISPRi essential calls that were not shared with TnSeq (n = 105), 53 (7.2% of all CRISPRi essential calls) were upstream of an essential gene in a potential operon (DeJesus et al., 2017), indicating that the CRISPRi essentiality call is likely explained by a polar effect. 30 remaining discrepant calls have been reported to be essential in some TnSeq screens (Griffin et al., 2011; Sassetti et al., 2003; Zhang et al., 2012), suggesting that these genes may indeed be essential but difficult to call by TnSeq or conditionally essential. The remaining 22 discrepant calls likely reflect differences in medium composition (Gandotra et al., 2007) between this CRISPRi experiment and TnSeq experiments or technical limitations of either approach for specific genes, such as transposon insertions in a non-essential domain of an otherwise essential gene (DeJesus et al., 2017). The broad overlap in essential gene calls between CRISPRi and TnSeq demonstrates the robustness of genome-scale CRISPRi to identify growth phenotypes in Mtb.

### Features that dictate sgRNA strength

**Figure 2:**
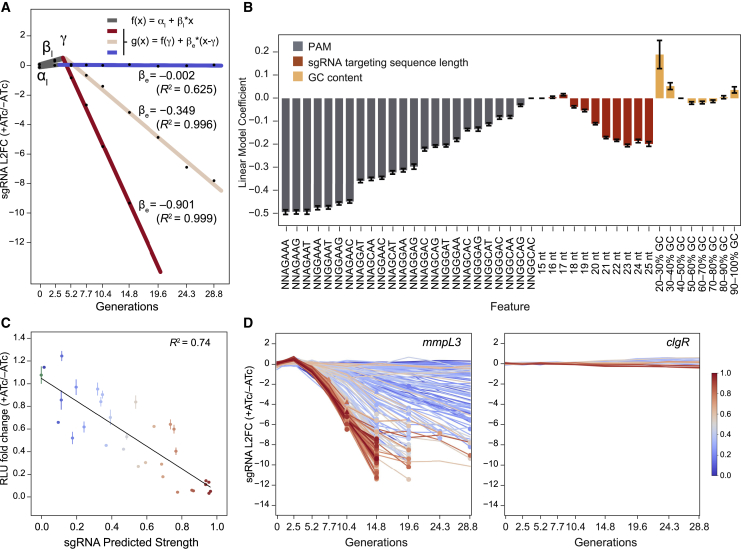
Identification of features that dictate sgRNA strength (A) Two-line model fits for three different sgRNAs targeting *mmpL3*. (B) Bar plot showing the regression coefficients (mean ± SEM) for each sgRNA feature identified by the linear model, colored by feature type. All features were represented by more than 500 sgRNAs except for the 20%–30% GC (n = 18) and 90%–100% GC (n = 458) bins. (C) Comparison of measured versus linear model predicted CRISPRi activity (mean ± SEM) of 29 sgRNAs against a *Renilla* luciferase target in Msmeg; sgRNAs are color coded from blue (strength = 0) to red (strength = 1). The green dot indicates a control non-targeting sgRNA. RLU, relative light unit. (D) Line plot showing the behavior of sgRNAs targeting the essential gene *mmpL3* and non-essential gene *clgR*. sgRNAs are color coded by predicted strengths as in (C). Circles represent our sequencing limit of detection. Triangles represent the point of observation of rare CRISPRi-resistant subpopulations, beyond which sgRNA L2FC values are not plotted (see STAR Methods for details). See also Figure S1 and Data S1.

To determine gene level vulnerability, we next needed to reliably predict sgRNA strength. We hypothesized that we could quantitatively define the features that control sgRNA strength by modeling the sgRNA growth behaviors observed in the CRISPRi screen. We first computed the fitness cost imposed by individual sgRNAs by fitting a piecewise linear regression model (hereafter referred to as a “two-line model”) to the sgRNA L2FC values (Figure 2A; Data S1). This model allowed us to study two distinct phases of sgRNA behavior, a phenotypic lag phase and a phase of sgRNA depletion along with the transition point (γ) between the two phases, and to exclude rare instances of CRISPRi-resistant cell subpopulations (Figures S1K–S1M; Data S1). The rate of depletion $\left(\beta_{e}\right)$ represents the fitness cost imposed by each sgRNA and was used to estimate sgRNA strength (Figure 2A).

We then applied a linear model to determine which sgRNA features were most important in predicting the rate of sgRNA depletion. Consistent with previous publications, we found that the PAM (Rock et al., 2017), sgRNA targeting sequence length (Qi et al., 2013), and GC content (Gilbert et al., 2014; Hawkins et al., 2020) contributed to sgRNA strength (Figure 2B; Figures S1N and S1O; Data S1).

To validate the sgRNA strength predictions, we designed sgRNAs of varying predicted strengths and measured how strongly they reduced expression of a luminescent reporter gene (*Renilla* luciferase) in Msmeg. We found a strong correlation (*R*^*2*^ = 0.74) between predicted sgRNA strength and *Renilla* knockdown (Figure 2C). That the linear model was trained on fitness phenotypes in Mtb and accurately predicted *Renilla* knockdown values in Msmeg (Figure 2C) further supports the hypothesis that CRISPRi knockdown efficacy is, at least in part, determined by biophysical parameters of the dCas9-sgRNA-DNA interaction. We then normalized sgRNA strength predictions to span values from 0 (weakest, blue) to 1 (strongest, red). The growth effects for sgRNAs of varying predicted strengths targeting an essential (*mmpL3*) and non-essential (*clgR*) gene (Figure 2D) generally matched the expected phenotypes, further demonstrating the broad tunability of target gene knockdown with this CRISPRi system.

### Bayesian modeling to quantify gene vulnerability

**Figure 3:**
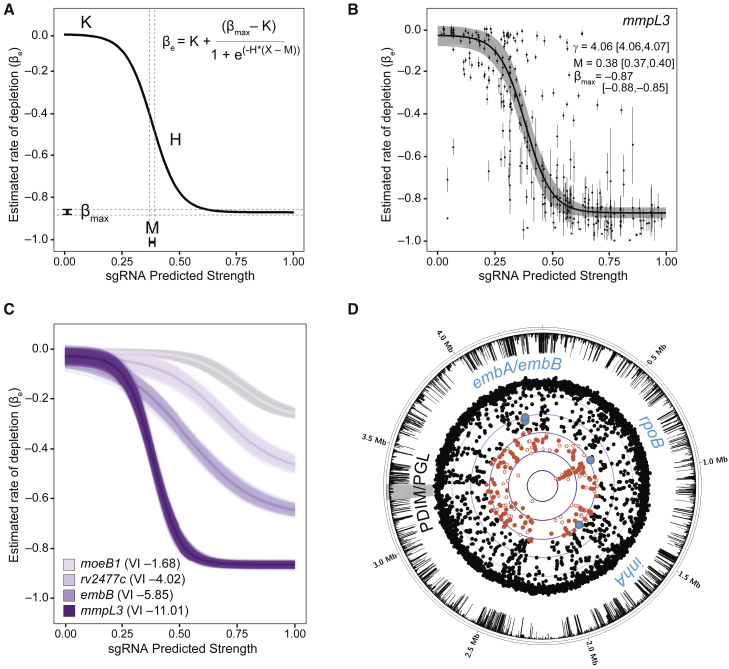
A quantitative framework to predict gene vulnerability to transcriptional silencing (A) Description of the logistic curve parameters used to model gene level vulnerability. The x axis depicts the linear model predicted strengths of gene-targeting sgRNAs. The y axis depicts the fitness cost of individual sgRNAs estimated by the two-line model from Figure 2A. See details in STAR Methods. (B) Logistic curve fit to all sgRNAs (dots) targeting *mmpL3*. The black line represents the mean logistic curve and range (gray) from 5,000 parameter samples. Mean parameter estimates and their 95% highest density interval (HDI) are indicated. (C) Logistic regression fits for four example genes of differing vulnerability along with their corresponding VI. Lines represent fits generated by the sampling procedure with the dark line representing the mean fit. (D) Circos plot showing all targeted Mtb H37Rv genes (dots) with their VI. Genes in the upper quartile of vulnerability are depicted as red dots (filled red, confident VI; unfilled red, low-confidence VI). Genes encoding the targets of first line TB therapy (*rpoB*, *inhA*, and *embAB*) are highlighted by blue dots. The outer ring represents the gene-level L2FC value at 28.8 generations. The inner purple lines represent decreasing VI values, with the most vulnerable genes located closest to the center of the circle. The phthiocerol dimycocerosates (PDIM)/phenolic glycolipid (PGL) locus (gray) contains no vulnerable genes. See also Figure S2 and Data S2.

**Figure S2:**
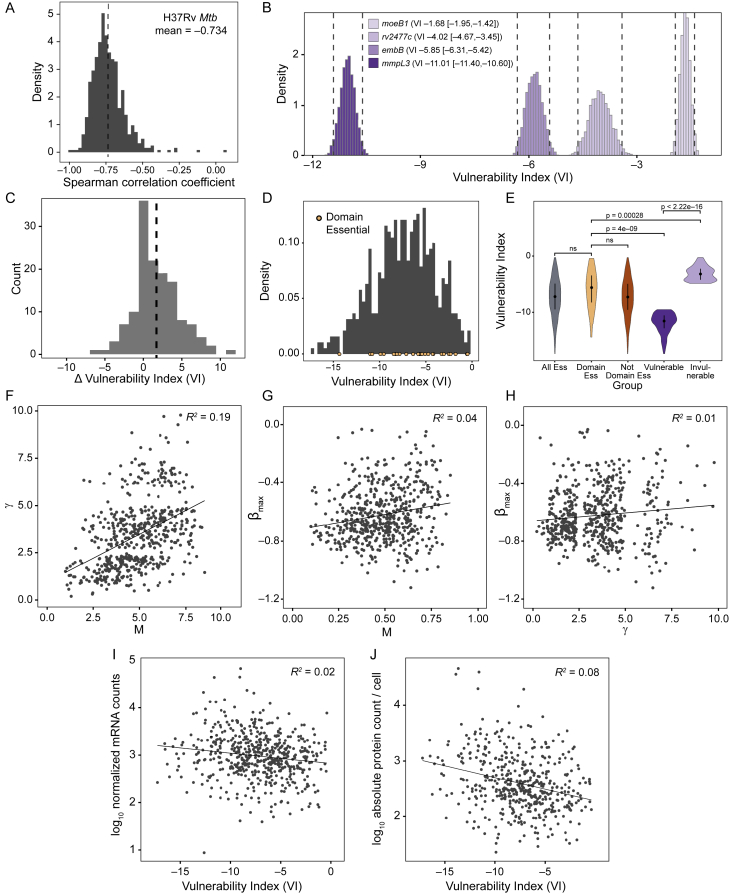
Individual vulnerability model parameters are gene specific, and vulnerability is not correlated with gene expression levels, related to Figure 3 and Data S1 (A) Histogram showing the per-gene Spearman correlation between the rate of depletion $\left(\beta_{e}\right)$ estimated from the Bayesian vulnerability model and the predicted strength for targeting sgRNAs. All CRISPRi essential genes with confident vulnerability calls in H37Rv (n = 552) are included in this analysis. (B) Histogram of the vulnerability indices estimated from 5,000 parameter samples for *mmpL3* (*rv0206c*), *embB* (*rv3795*), *rv2477c*, and *moeB1* (*rv3206c*). The vulnerability index 95% credible regions are depicted by dashed lines. (C) Histogram showing the potential influence of the CRISPRi polar effect on vulnerability. The difference in vulnerability index between any downstream gene and its respective upstream gene in the operon is depicted (VI downstream gene – VI upstream gene; n = 657 comparisons). Dashed line depicts the mean difference in VI (mean, 1.658). (D) Histogram of vulnerability indices for genes predicted to be essential by CRISPRi and with confident vulnerability calls, highlighting genes predicted to have an essential domain according to TnSeq (DeJesus et al., 2017). (E) Violin plot depicting the vulnerability index for different groups of genes: all CRISPRi essential genes with confident vulnerability calls (All Ess; n = 552), genes predicted to have an essential domain (Domain Ess; n = 26), genes without an essential domain (Not Domain Ess; n = 526), and genes in the top (n = 138) and bottom (n = 138) quartiles of vulnerability index. Dot and error bars represent mean and SD. Significance (p-value) is calculated using a two-sided t-test. (F-H) Scatterplot of gene vulnerability ratios and/or individual gene parameter estimates. Only confident vulnerability index estimates are shown (see main text for details). (F) depicts the relationship between γ and M; (G) depicts the relationship between $\beta_{max}$ and M; (H) depicts the relationship between $\beta_{max}$ and γ. (I and J) Scatterplot showing the relationship between gene mRNA levels as quantified by RNaseq (I) or protein levels as quantified by mass spectrometry (J) (Schubert et al., 2015) and gene vulnerability. Only confident vulnerability index estimates are shown (see STAR Methods for details).

Having generated reliable sgRNA strength predictions, we next sought to integrate these into a gene level estimate of vulnerability. We used a Bayesian multilevel model to capture the relationship between the magnitude of target knockdown, as estimated from predicted sgRNA strength, and the resulting fitness cost to the bacterium. The model includes an “sgRNA level,” defined by the two-line model described in Figure 2A, and a “gene level” based on a logistic curve (Figure 3A) described by four parameters: the minimum gene level fitness cost (K), the maximum fitness cost $\left(\beta_{max}\right)$, the predicted sgRNA strength at which fitness cost reaches the mid-point (M), and the Hill coefficient (H). The phenotypic lag between CRISPRi activation and observed fitness defects for a gene (i.e., gene level γ) is estimated from the mean γ of the individual gene-targeting sgRNAs. Figure 3B depicts the logistic curve fit for *mmpL3*. Importantly, we found a strong per-gene Spearman correlation (mean, –0.734) between the predicted strength of each sgRNA and $\beta_{e}$ estimated by the vulnerability model (Figure S2A), further validating the sgRNA strength predictions.

To summarize gene vulnerability into a single quantitative metric, we then integrated the predicted fitness costs for sgRNAs spanning the sgRNA strength range (0–1) for each gene. To do this, we used Bayesian multilevel model fits (Figure 3A) when needed to impute the behavior of all possible sgRNA strengths not measured in our CRISPRi library. The total fitness cost associated with all theoretical sgRNAs was summed into one value, which we refer to as the “vulnerability index” (VI) or gene vulnerability. To ensure robust vulnerability calls, we focused on essential genes that were experimentally targeted with sgRNAs of a wide range of strengths and had highly consistent parameter estimates. This filtering resulted in confident vulnerability assessments for ∼93% of all TnSeq essential genes (n = 580 of 624).

We found that vulnerability varies widely across the Mtb genome (Figures 3C and 3D; Figure S2B; Data S2). Genes encoding the targets of the two most potent first-line TB drugs, isoniazid (*inhA*) and rifampicin (*rpoB*), were among the upper quartile of vulnerable genes, although many considerably more vulnerable genes exist. We found the first gene in an operon containing essential genes to have modestly increased vulnerability in 65% of candidate operons (n = 55 of 85), demonstrating that the CRISPRi polar effect is not a primary driver of the VI (Figure S2C). Domain essential genes as defined by TnSeq (DeJesus et al., 2017) did not have significantly different vulnerabilities compared with the average essential gene (Figures S2D and S2E), excluding domain essentiality as a primary driver of VI. There was little correlation between the gene level γ, M, and $\beta_{max}$ parameters, demonstrating that individual genes have widely varying combinations of each parameter (Figures S2F–S2H). VI was also not correlated with the targets’ mRNA or protein levels (Figures S2I and S2J). Thus, the most vulnerable processes under these growth conditions are not necessarily the most highly expressed.

### Validation of vulnerability predictions

Our vulnerability estimates are predicated on the correlation between predicted sgRNA strength and the magnitude of target knockdown. Visual analysis of sgRNA level fitness effects (Figure 2D), strong per-gene correlation between the predicted strength of each sgRNA and $\beta_{e}$ (Figure S2A), and direct quantification of this correlation against the exogenous *Renilla* gene (Figure 2C) are largely consistent with this hypothesis. To further test this fundamental hypothesis, we sought to quantify the magnitude of target knockdown at a set fitness cost for genes of varying vulnerability. On average, more vulnerable genes should require lower levels of inhibition than invulnerable genes to achieve the same fitness cost.

**Figure S3:**
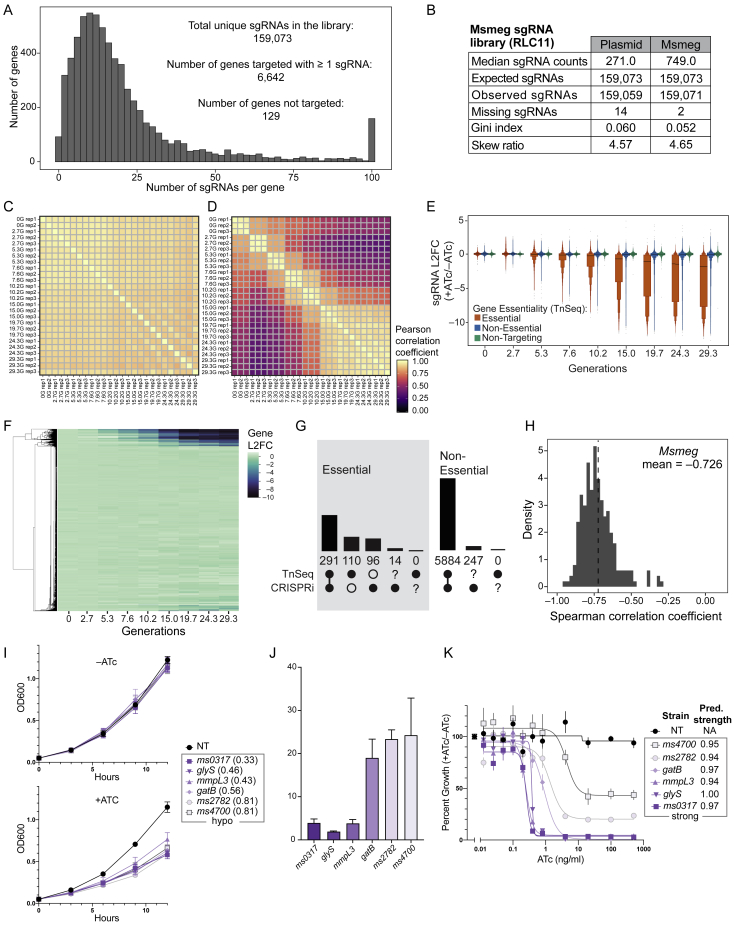
Genome-scale CRISPRi in Msmeg, related to Figure 4, Table S1, and STAR Methods (A) Histogram depicting the number of sgRNAs per gene in the Msmeg CRISPRi library (RLC11; Addgene #163955). The library targets 6,642 of the 6,679 annotated Msmeg genes. (B) Next generation sequencing quality-control metrics for the Msmeg CRISPRi library. The “Plasmid” column depicts metrics for the RLC11 plasmid library following cloning and isolation from *E. coli*. The “Msmeg” column depicts library metrics following transformation and expansion in Msmeg. Skew ratio represents the ratio between top and bottom 10% of sgRNA counts. (C and D) Correlation heatmap of the triplicate screens performed in Msmeg depicting TnSeq essential gene (Dragset et al., 2019) targeting sgRNAs in the –ATc (C) and +ATc (D) cultures. (E) Boxen plots comparing time-dependent changes in sgRNA L2FC values targeting genes defined as Essential (n = 27,702 sgRNAs) and Non-Essential (n = 120,429) by TnSeq (Dragset et al., 2019). Mean L2FC (solid line) and quantiles beyond the 25^th^ and 75^th^ percentiles are shown (boxes). Also depicted are control Non-Targeting sgRNAs (n = 7,421). (F) Hierarchical clustering of gene level depletion from the Msmeg CRISPRi fitness screen. Each row represents a single targeted Msmeg gene. (G) Bar chart showing the overlap between gene calls by TnSeq (Dragset et al., 2019) and CRISPRi. 73% of TnSeq essential calls (291 of 401) are shared with CRISPRi. (H) Histogram showing the per-gene Spearman correlation between the rate of depletion $\left(\beta_{e}\right)$ estimated from the Bayesian vulnerability model and the predicted strength for targeting sgRNAs. All CRISPRi essential genes with confident vulnerability calls in Msmeg are included in this analysis. (I) Growth kinetics of the hypomorphic sgRNAs (mean ± SD) shown in Figure 4C. The linear model predicted sgRNA strengths are listed in parentheses next to each gene name. All strains were grown for 15 generations in the presence or absence of ATc and then used to seed cultures for the time-course experiment shown here. Growth for 15 generations ± ATc ensures all strains have reached steady-state growth in response to CRISPRi target gene knockdown. NT, non-targeting. (J) Quantification of target gene mRNA levels by qRT-PCR (biological triplicates; mean ± SEM) of the hypomorphic strains depicted in Figure 4C. (K) Effect of titrating the ATc concentration (range 0-500 ng/mL) on growth (mean ± SD) of the indicated strains from Figure 4C. These strains encode either a non-targeting (NT) sgRNA or a strong sgRNA (predicted strength range, 0.94 – 1.00) against the indicated target. Strains are color coded by vulnerability as in Figure 4D.

**Figure 4:**
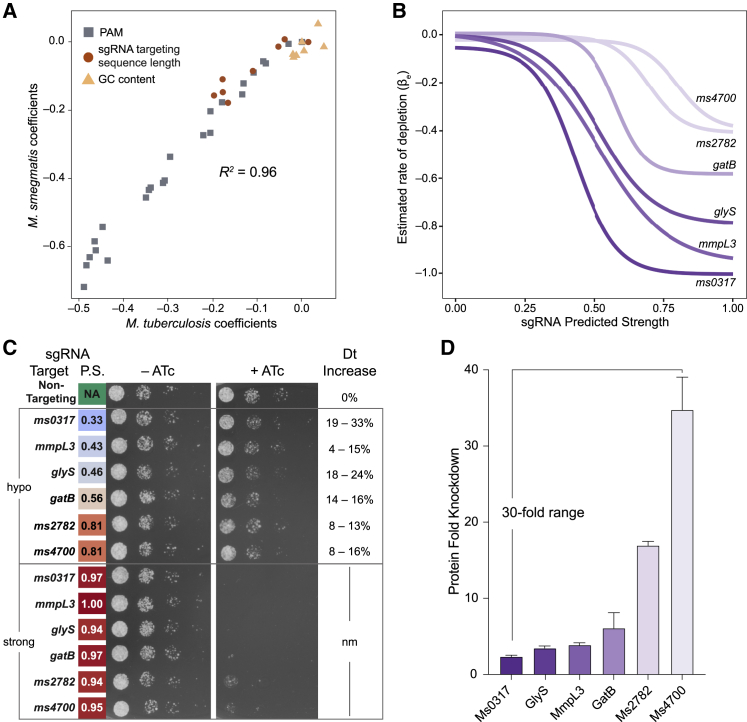
Vulnerability predictions correlate with the magnitude of target knockdown needed to reduce bacterial fitness (A) Scatterplot of the linear model coefficients (as in Figure 2B) for Mtb H37Rv (x axis) and Msmeg (y axis). (B) Mean logistic regression fits for the indicated Msmeg genes of varying vulnerability. (C) Phenotypic consequences of hypomorphic (hypo) and strong knockdown of the genes depicted in (B). Predicted sgRNA strengths (P.S.) are listed next to each sgRNA and are color coded according to the scale in Figure 2D. The percent increase in strain doubling time (Dt) of each hypo sgRNA compared with a non-targeting control (95% confidence interval [CI]) was quantified at steady-state growth (Figure S3I). nm, not measured. (D) Quantification of target gene protein levels (mean ± SD) by label-free mass spectrometry (+ATc) of the 6 hypo strains depicted in (C). qRT-PCR quantification of target gene mRNA levels for the same strains is depicted in Figure S3J. See also Figure S3, Table S1, and Data S2.

To facilitate testing this hypothesis, we turned to Msmeg. We first performed a genome-scale CRISPRi experiment similar to that described in Figure 1A (detailed in Figures S3A and S3B). Growth phenotypes were well correlated among triplicate screens (Figures S3C and S3D). Application of the linear model (Figure 2B) to generate sgRNA strength predictions produced highly concordant results (*R*^*2*^ = 0.96) between Msmeg and Mtb (Figure 4A). This CRISPRi screen produced gene essentiality calls that were broadly consistent (73% overlap) with a recently published Msmeg TnSeq dataset (Dragset et al., 2019; Figures S3E–S3G). As in Mtb, we found a strong per-gene Spearman correlation (mean, –0.726) between the sgRNA predicted strength and $\beta_{e}$ (Figure S3H). After quantifying vulnerability for all Msmeg genes (Data S2), we identified six essential genes for follow-up: *ms0317*, *mmpL3* (*ms0250*), *glyS* (*ms4485*), *gatB* (*ms2367*), *ms2782*, and *ms4700*. We selected these genes because of their wide range in predicted VI (Figure 4B), the absence of a potential polar effect, and the existence of an annotated Mtb homolog of similar vulnerability. For each target we then designed two sgRNAs: one strong sgRNA predicted to result in high-level gene knockdown and one hypomorphic sgRNA expected to result in partial gene knockdown and a similar growth defect for all six targets. Consistent with all six genes being essential in Msmeg, the strong sgRNA prevented growth for all targeted genes (Figure 4C). All hypomorphic sgRNAs produced similar fitness costs (∼10%–30% increase in doubling time; Figure 4C; Figure S3I). We then determined the magnitude of target knockdown for all six targets under partial gene silencing. In agreement with our predictions, there was an inverse correlation between vulnerability and the magnitude of target mRNA and protein knockdown required to impose the same fitness cost (Figure 4D; Figure S3J), spanning more than a 30-fold range between the most vulnerable gene and invulnerable gene tested (Figure 4D). Last, we validated these results by tuning the magnitude of target knockdown by varying ATc concentrations for the six Msmeg strains harboring strong sgRNAs (Vigouroux et al., 2018). Genes predicted to be more vulnerable showed a lower ATc minimum inhibitory concentration (MIC) than invulnerable genes, again indicating that vulnerable genes, on average, require lower levels of transcriptional inhibition to impose a fitness cost (Figure S3K). These results validate that our CRISPRi-based method to quantify gene vulnerability relates the magnitude of target knockdown and resulting strain fitness.

### Evolutionary conservation of vulnerability

**Figure 5:**
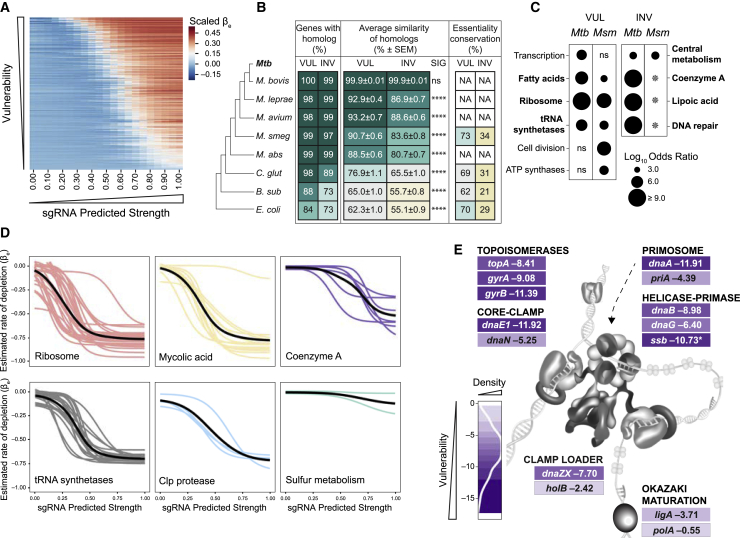
Pathway analysis identifies differentially vulnerable processes in mycobacteria (A) Heatmap of fitness cost (scaled $\beta_{e}$) as a function of increasing sgRNA strength. Each row represents a single Mtb gene for which a high-confidence VI is available. (B) Table depicting evolutionary conservation between Mtb and eight other bacterial species. For the most vulnerable (VUL; n = 138) and invulnerable (INV; n = 138) H37Rv Mtb genes, the frequency with which a homolog was identified (“genes with homolog”) and the average amino acid similarity (“average similarity of homologs”; % ± SEM) are reported. For the four bacterial species for which genome-wide essentiality calls are available, conservation of essentiality (%) is also listed. *M. smeg*, *M. smegmatis*; *M. abs*, *M. abscessus*; *C. glut*, *C. glutamicum*; *B. sub*, *B. subtilis*. ^∗∗∗∗^p < 0.0001. ns, not significant. (C) Bubble plot of the enriched (p < 0.05) PATRIC subclasses for the top quartile VUL and bottom quartile INV Mtb and Msmeg (*Msm*) genes. Conserved subclass enrichment is depicted in bold type. The star represents subclasses where some or all of the corresponding Msmeg homologs are non-essential (Figure S4C), which, for the purposes of this analysis, were considered INV. (D) Logistic regression curves of the indicated Mtb gene groups. Each colored line represents a single gene. The solid black line represents the locally estimated scatterplot smoothing (LOESS) fit of the individual mean logistic regressions. (E) Detailed view of the different vulnerabilities of Mtb genes involved in DNA replication. Genes are color coded by their VI. Darker shades of purple indicate higher vulnerability. The density scale represents the fraction of CRISPRi essential genes with confident VI calls. Figure adapted from (Yao and O’Donnell, 2010). ^∗^, low-confidence call. See also Figures S4 and S5 and Data S2.

**Figure S4:**
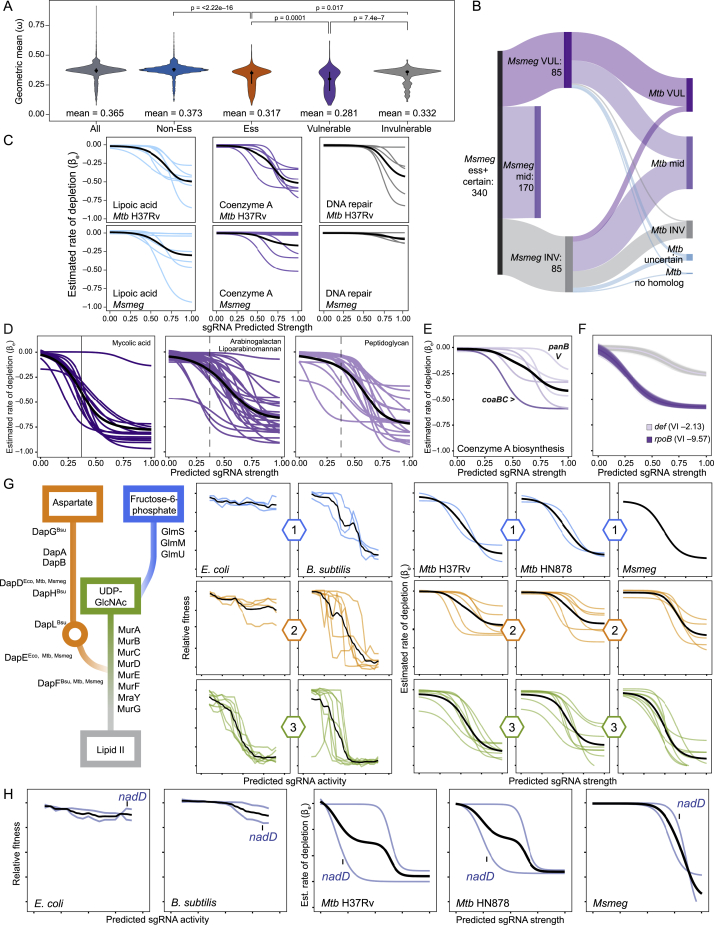
Evolutionary conservation of vulnerability, related to Figure 5 and Data S2 (A) Violin plot depicting the gene level dN/dS ratios (ω) estimated by GenomegaMap (Wilson and CRyPTIC Consortium, 2020) for five groups of genes: all analyzed Mtb genes (All; n = 3,979), TnSeq non-essential genes (Non-Ess; n = 3,271), TnSeq essential genes (Ess; n = 624), and genes in the top (Vulnerable, n = 138) and bottom (Invulnerable; n = 138) quartiles of vulnerability index. Dot and error bars represent mean and SD. Significance (p-value) is calculated with a two-sided t-test. (B) Sankey plot showing vulnerability conservation between Msmeg and Mtb. 92% of (78 of 85) vulnerable Msmeg genes (VUL; upper quartile) have an Mtb homolog that also ranks in the upper or middle quartile of vulnerability. 82% (70 of 85) of the invulnerable Msmeg genes (INV; lower quartile) have an Mtb homolog that ranks in the lower or middle quartile of vulnerability. (C) Logistic regression curves of the indicated Mtb H37Rv and Msmeg gene groups (PATRIC subclasses indicated above the species name) starred in Figure 5C. Each colored line represents the mean logistic regression curve for a single gene. The solid black line represents the LOESS fit of all logistic regressions. Note that several invulnerable Mtb genes are non-essential in Msmeg. (D) Logistic regression fits and summary LOESS fit for the indicated gene groups (PATRIC subclass) that synthesize the three main mycobacterial cell envelope components. The dashed line is a reference to the solid line of the mycolic acid biosynthesis genes. (E) Vulnerability estimates for the Mtb coenzyme A biosynthetic pathway. Genes are color coded as in Figure 5E. Shown are the mean logistic regression curves for each gene. The solid black line represents the LOESS fit for the indicated gene group. (F) Logistic regression fits for the drug targets *rpoB* (*rv0667*) and *def* (*rv0429c*). Lines represent fits generated by the sampling procedure with the dark line representing the mean fit. (G) Expression-fitness relationships for genes involved in the cytoplasmic steps of peptidoglycan synthesis between *E.coli*, *B. subtilis* (adapted from Hawkins et al., 2020), and Mtb H37Rv, Mtb HN878 and Msmeg. Each colored line represents the expression-fitness relationship for a single gene in the indicated group; the solid black line represents the LOESS fit for the indicated gene group. Only genes with confident vulnerability calls are shown for Mtb and Msmeg. (H) Comparison of the expression-fitness relationships of *nadD* and *nadE* between *E.coli*, *B. subtilis* (adapted from Hawkins et al., 2020), and Mtb H37Rv, Mtb HN878 and Msmeg.

We next sought to understand the natural selection pressures operating on vulnerable and invulnerable genes. We first ranked genes based on their VI in H37Rv Mtb (Figure 5A). We then compared the non-synonymous to synonymous substitution ratio (dN/dS or ω) estimates from 10,209 Mtb whole-genome sequences (Wilson and CRyPTIC Consortium, 2020). Consistent with previous reports (Comas et al., 2010), we found that essential Mtb genes are, on average, under higher purifying selection than non-essential genes (p < 2.22e−16) (Figure S4A). Highly vulnerable genes had significantly lower dN/dS ratios than all essential genes (p = 0.0001), whereas invulnerable genes had higher dN/dS ratios (p = 0.017) (Figure S4A). These results provide an orthogonal metric distinguishing these two gene sets. We next investigated conservation of these gene sets across eight bacterial species spanning more than 2 billion years of evolution. We found that vulnerable Mtb genes are more likely to have a homolog in other bacterial species than invulnerable genes (Figure 5B). Moreover, when a homolog exists, vulnerable gene homologs are more highly conserved and more likely to be essential in other bacterial species (Figure 5B). Thus, vulnerable genes are more conserved and evolutionarily constrained than invulnerable genes.

### Pathway analysis of vulnerability

**Figure S5:**
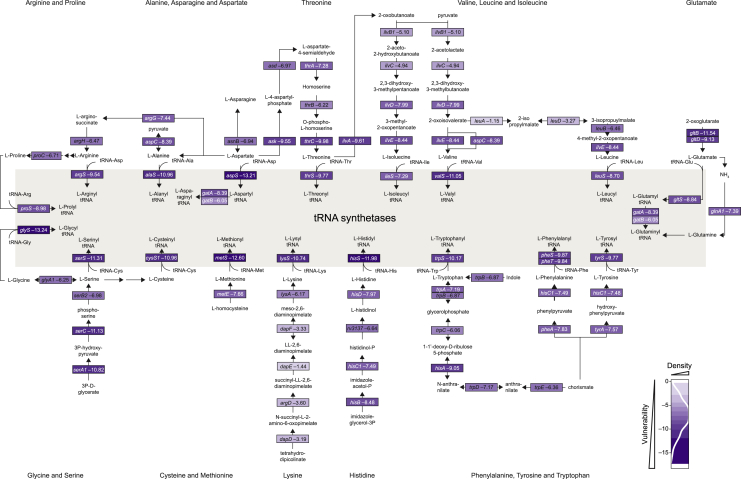
tRNA synthetases are choke points in Mtb translation, related to Figure 5 and Data S2 Vulnerability estimates for Mtb H37Rv amino acid metabolic genes and tRNA synthetases. Only genes that are CRISPRi essential and have a vulnerability call with high confidence are shown. Genes are color coded as in Figure 5E. The density scale in the figure legend represents the fraction of CRISPRi essential genes with certain vulnerability calls.

Having defined target vulnerability at genome scale, we next performed pathway enrichment analysis for the most vulnerable and invulnerable genes in H37Rv Mtb and Msmeg (Figure 5C). Consistent with the evolutionary conservation identified in Figure 5B, we identified substantial but incomplete overlap between enriched pathways in H37Rv Mtb and Msmeg (Figure 5C), and the invulnerable gene set had more discrepant vulnerability classifications than the vulnerable gene set (Figures S4B and S4C). As expected, central dogma processes are enriched for vulnerable genes. This includes protein translation (Figures 5C and 5D), consistent with work in other bacteria demonstrating a linear relationship between growth rate and the number of ribosomes per cell (Scott et al., 2010). Intriguingly, tRNA synthetases as a class are universally vulnerable (Figures 5C and 5D), whereas amino acid biosynthesis is less vulnerable (Figure S5), highlighting tRNA synthetases as a choke point in Mtb translation. Although transcription is enriched as a subclass in the most vulnerable gene set (Figure 5C), DNA replication is unexpectedly not. This apparent discrepancy can be explained by the large variability in VI of genes essential for DNA replication in Mtb. Although genes like the replicative polymerase *dnaE1* and the gyrase subunits *gyrA* and *gyrB* are highly vulnerable, genes involved in Okazaki fragment maturation (*polA* and *ligA*) are comparatively invulnerable (Figure 5E). The invulnerability of Okazaki fragment maturation raises concerns about its attractiveness as a drug target (Reiche et al., 2017). We further find that genes in the “fatty acids” category are almost universally vulnerable, consistent with the wide variety of whole-cell active compounds discovered to inhibit mycolic acid biosynthesis (Figures 5C and 5D). At the pathway level, mycolic acid biosynthesis is more vulnerable than other envelope biogenesis pathways (Figure S4D), although some targets within peptidoglycan (*murB* and *murX*) and arabinogalactan biosynthesis (*ubiA*, *glfT2*, *glf*, and *dprE1*) are highly vulnerable. Numerous additional processes beyond those expected to be critical for rapid growth are found in the most vulnerable gene set, including the Clp protease complex (Figure 5D), a target currently under intense investigation in TB drug discovery (Lupoli et al., 2018), and enzymes involved in protein folding, protein secretion, cell division, and energy metabolism (Data S2).

We found that metabolic processes such as central metabolism, lipoic acid (an essential post-translational modification critical for some central metabolic enzymes), and sulfur metabolism were enriched in the invulnerable gene set (Figures 5C and 5D), consistent with some metabolic enzymes being maintained at higher levels than required to maintain metabolic flux (Donati et al., 2018). Coenzyme A biosynthesis is also invulnerable (Figures 5C and 5D), potentially providing an explanation for the failure to develop drugs that inhibit Mtb CoaA (Barry et al., 2009). Consistent with published results, we identified *coaBC* as a choke point within coenzyme A biosynthesis (Evans et al., 2016; Figures S4C and S4E), although this gene nevertheless remains less vulnerable than targets in more vulnerable pathways. Last, we find that peptide deformylase *def* (*rv0429c*) is highly invulnerable (Figure S4F), again potentially explaining the failure to advance hit compounds against this intensely pursued target (Barry et al., 2009).

We next expanded our vulnerability conservation analysis to *E. coli* and *B. subtilis*, which have been profiled recently by a mismatched CRISPRi approach to examine expression-fitness relationships (Hawkins et al., 2020). Despite methodological differences, all four bacterial species showed similar vulnerability profiles for genes involved in translation (vulnerable) and cofactor biosynthesis (generally invulnerable). However, there were notable differences between the species. The vulnerability profiles of peptidoglycan precursor biosynthetic enzymes were more similar between the Gram-positive *B. subtilis* and mycobacteria than with the Gram-negative *E. coli* (Figure S4G). Although peptidoglycan biosynthesis was highly vulnerable in *B. subtilis*, as noted above, it is less vulnerable than mycolic acid biosynthesis in mycobacteria (Figure S4D). Furthermore, the last two steps of nicotinamide adenine dinucleotide (NAD) biosynthesis mediated by *nadD* and *nadE* are much more vulnerable in mycobacteria than *E. coli* and *B. subtilis* (Figure S4H). This may be explained by the lack of a functional *nadR* salvage pathway in Mtb (Boshoff et al., 2008; Rodionova et al., 2014), which results in all NAD biosynthetic flux through *nadDE*. The remarkable vulnerability of *nadD* relative to *nadE* in Mtb suggests that NadD might be rate limiting for NAD synthesis or have a moonlighting function in another process. These results illustrate the potential to pursue conserved, vulnerable processes as targets of broad-spectrum antibiotics (e.g., translation) as well as more narrow-spectrum agents (e.g., mycolic acid biosynthesis).

This analysis highlights the ability of a genome-scale vulnerability assessment to identify rate-limiting steps in Mtb physiology, validate known and nominate new targets for drug discovery, and provide potential explanations for previously failed drug discovery efforts.

### Conservation of vulnerability in the hypervirulent Mtb strain HN878

Our studies to this point used the reference Mtb strain H37Rv, a lineage 4 strain (Cole et al., 1998). A growing body of evidence demonstrates heterogeneity among Mtb strains in clinically relevant characteristics, including response to antibacterial agents (Carey et al., 2018; Coscolla and Gagneux, 2010). Thus, we also assessed target vulnerability in the hypervirulent lineage 2 Mtb clinical isolate HN878 (Sreevatsan et al., 1997).

**Figure S6:**
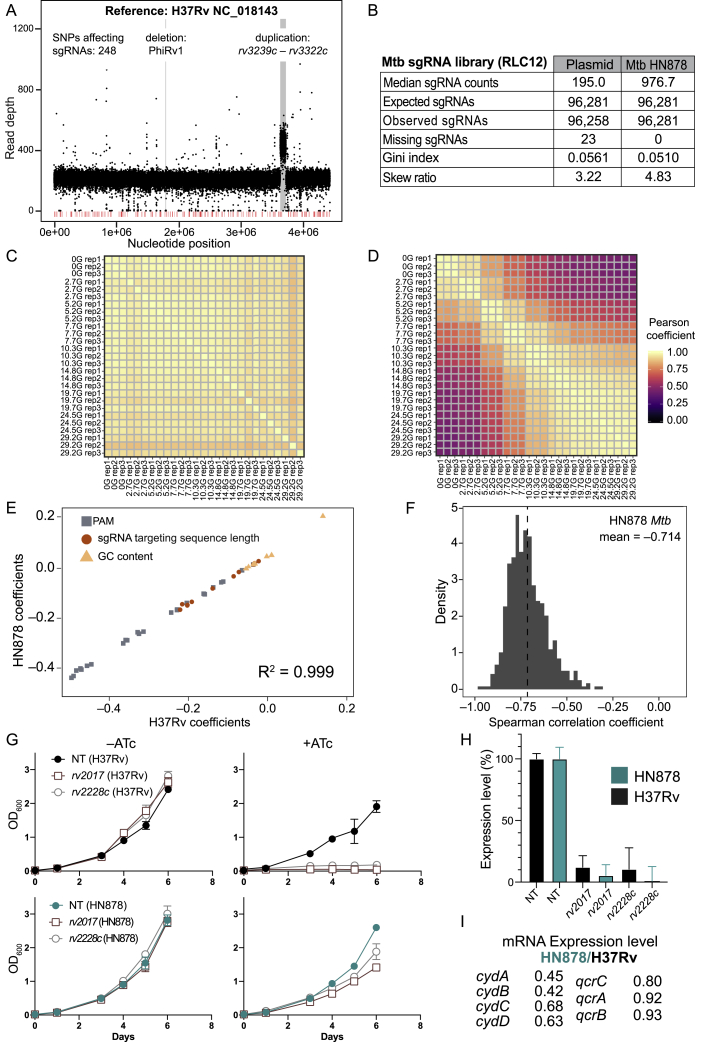
Genome-scale CRISPRi in Mtb HN878, related to Figure 6 and STAR Methods (A) Read depth plot of Mtb HN878 whole genome sequencing mapped to the H37Rv genome (GenBank: NC_018143). The 248 SNPs affecting 664 sgRNAs of our CRISPRi library are indicated in red. Significant decreases and increases in read depth mark a genomic deletion and duplication, respectively, in our HN878 clone and are highlighted in gray. (B) Next generation sequencing quality-control metrics for the Mtb HN878 CRISPRi library. The “Plasmid” column depicts metrics for the RLC12 plasmid library following cloning and isolation from *E. coli*. The “Mtb HN878” column depicts library metrics following transformation and expansion in Mtb HN878. Skew ratio represents the ratio between top and bottom 10% of sgRNA counts. (C) Correlation heatmap of the triplicate screens performed in Mtb HN878 depicting TnSeq essential gene (DeJesus et al., 2017) targeting sgRNAs in the –ATc cultures. (D) Correlation heatmap of the triplicate screens performed in Mtb HN878 depicting TnSeq essential gene (DeJesus et al., 2017) targeting sgRNAs in the +ATc cultures. (E) Scatterplot of the linear model coefficients (as in Figure 2B) for Mtb H37Rv (x axis) and Mtb HN878 (y axis). (F) Histogram showing the per-gene Spearman correlation between the rate of depletion $\left(\beta_{e}\right)$ estimated from the Bayesian vulnerability model and the predicted strength for targeting sgRNAs. All CRISPRi essential genes with confident vulnerability calls in HN878 are included in this analysis. (G) Liquid growth assay (mean ± SD) using the sgRNAs targeting two differentially essential genes used in Figure 6B. NT, non-targeting. (H) Quantification of target gene mRNA levels by qRT-PCR (n = 6 technical replicates; mean ± SEM) following CRISPRi silencing of *rv2017* and *rv2228c* in H37Rv and HN878. Gene expression levels were normalized to the non-targeting control for each strain. (I) Quantification of target gene mRNA levels by qRT-PCR (technical triplicates of biological duplicates) of *cydABCD* and *qcrCAB* in H37Rv and HN878. For each gene, HN878 expression levels were compared to H37Rv (control).

**Figure 6:**
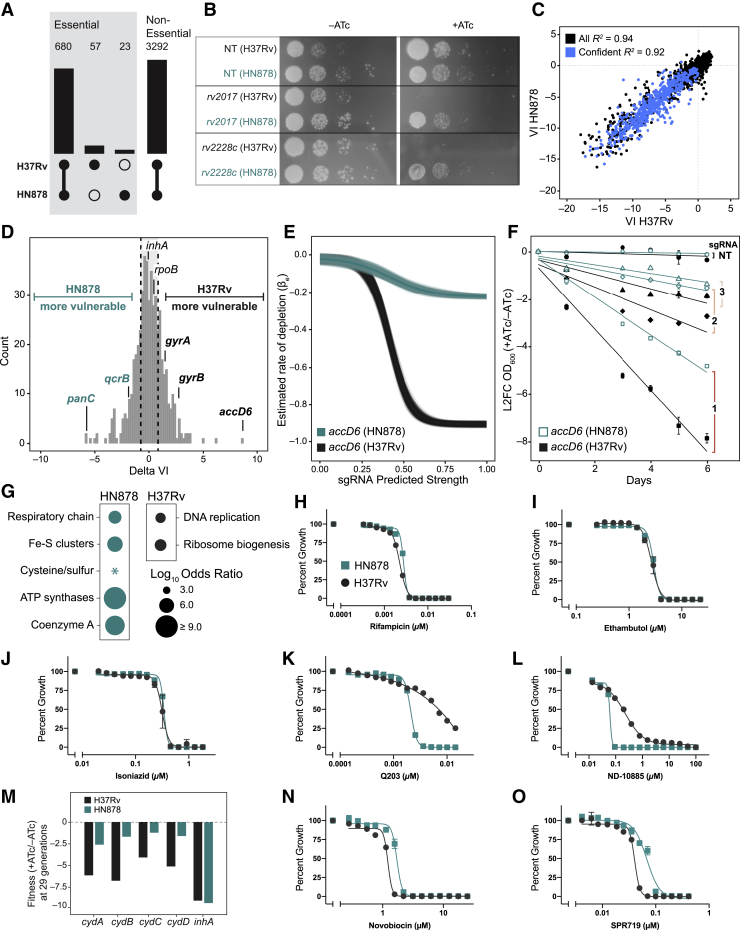
Differential VI predicts strain-specific susceptibility to antibacterial agents (A) Bar chart showing the overlap between CRISPRi gene essentiality calls in H37Rv and HN878. (B) CRISPRi knockdown of two genes predicted to be essential in H37Rv and non-essential in HN878. NT, non-targeting. (C) Correlation between VI in H37Rv and HN878 for all genes (black) and CRISPRi essential genes for which high-confidence VI calls are available (blue). (D) Histogram showing the normalized differential VI between HN878 and H37Rv for genes with a high-confidence call in both strains. Quartiles are delineated by a dotted line. (E) Logistic regression fits for *accD6* in H37Rv (black) and HN878 (turquoise). Lines represent fits generated by the sampling procedure with the dark line representing the mean fit. (F) Phenotypic consequences of *accD6* knockdown. The optical density 600 (OD_600_) L2FC (+ATc/–ATc; mean ± SD) was calculated for three *accD6* sgRNAs (1–3) and a non-targeting control sgRNA in H37Rv and HN878. Strains were pre-treated with ATc for 3 days prior to starting the depicted time course. (G) Bubble plot of the enriched (p < 0.05) PATRIC subclasses for genes more VUL in HN878 versus H37Rv. The star represents a subclass where some or all of the corresponding H37Rv homologs are non-essential, which, for the purposes of this analysis, were considered INV. (H–L) Effect of rifampicin (H), ethambutol (I), isoniazid (J), Q203 (K), and ND-10885 (L) on growth (mean ± SD) of H37Rv and HN878. (M) Gene-level L2FC measurements for *cydABCD* and *inhA* from the H37Rv and HN878 CRISPRi screens at ~29 generations. (N and O) Effect of novobiocin (N) and SPR719 (O) on growth (mean ± SD) of H37Rv and HN878. See also Figure S6 and Data S2.

Whole-genome sequencing of our HN878 clone identified 1,460 SNPs relative to H37Rv, 248 of which were predicted to affect only 664 sgRNAs of 96,700 in our library (Figure S6A). Thus, we transformed the same CRISPRi library used in H37Rv into HN878 and performed a competitive fitness experiment as in Figure 1A (Figure S6B). Growth phenotypes were well correlated among triplicates (Figures S6C and S6D). Linear model (Figure 2B) sgRNA strength predictions produced highly concordant results (*R*^*2*^ = 0.999) between H37Rv and HN878 (Figure S6E). As in H37Rv, we found a strong per-gene Spearman correlation between the predicted sgRNA strength and $\beta_{e}$ (Figure S6F). Although gene essentiality calls were largely conserved between the two strains, 80 genes were differentially essential (Figures 6A and 6B; Data S2). We confirmed *rv2017* and *rv2228c* as essential in H37Rv and dispensable in HN878 (Figure 6B; Figure S6G) and validated that this differential essentiality is not due to lack of CRISPRi targeting efficacy in either strain (Figure S6H).

Quantification of gene vulnerability revealed strong concordance between H37Rv and HN878 (*R*^*2*^ = 0.92), but this conservation was not universal (Figures 6C and 6D; Data S2). The pre-clinical drug target *accD6* (Reddy et al., 2014) was predicted to be the most differentially vulnerable gene (Figures 6D and 6E). We validated that CRISPRi inhibition of *accD6* produced a much larger fitness costs in H37Rv than in HN878 (Figure 6F). To determine the most differentially vulnerable pathways, we next performed pathway enrichment analysis (Figure 6G). Numerous components of cellular respiration and the electron transport chain were identified as significantly more vulnerable in HN878 (Figure 6G; Data S2), including the cytochrome *c* reductase encoded by *qcrCAB*. The differential VI of *qcrB* was somewhat unexpected, given previous reports demonstrating similar susceptibilities between H37Rv and HN878 to preclinical QcrB inhibitors (Lupien et al., 2020), but consistent with reports of other Mtb clinical isolates being more susceptible to QcrB inhibition (Arora et al., 2014). We thus determined the sensitivities of H37Rv and HN878 to a panel of different antibacterial agents targeting QcrB and control proteins encoded by genes of similar vulnerability. Consistent with the vulnerability predictions, HN878 was much more sensitive than H37Rv to two different QcrB inhibitors: HN878 demonstrated full growth inhibition upon treatment with QcrB inhibitors, whereas H37Rv outgrew over the course of the experiment (Figures 6H–6L). The MICs for the control drugs were similar between HN878 and H37Rv (Figures 6H–6L). We hypothesized that HN878 may preferentially use cytochrome *bc1-aa3* as opposed to cytochrome *bd* as the terminal oxidase of the electron transport chain. Consistent with this hypothesis, HN878 was more fit than H37Rv upon CRISPRi inhibition of cytochrome *bd* (Figure 6M) and expressed modestly lower levels of *cydA* and *cydB* than H37Rv (Figure S6I), as also observed in other Mtb clinical strains (Arora et al., 2014). Last, pathway enrichment analysis indicated that DNA replication was more vulnerable in H37Rv, driven in part by increased vulnerability of *gyrAB* in H37Rv (Figures 6D and 6G). To test this prediction, we compared the sensitivities of H37Rv and HN878 to two DNA gyrase inhibitors, novobiocin and SPR719 (Locher et al., 2015). We found that H37Rv is indeed more sensitive to novobiocin and SPR719 than HN878 (Figures 6N and 6O). This analysis highlights the capability of our approach to reveal differential genetic requirements between Mtb strains. These differences may be mediated by activity-modifying SNPs within the differentially essential/vulnerable genes or within non-target genes that influence gene or pathway activity and are an important area for follow-up study.

## Discussion

New approaches are needed to quantitatively describe gene essentiality to better understand microbial physiology and advance drug discovery. To address this challenge, we developed a CRISPRi-based functional genomics method capable of systematically titrating the expression of nearly all genes in two species of mycobacteria and monitoring the resulting effect on bacterial fitness. We found that essential genes exist along a gradient of vulnerability across and within pathways.

Our approach builds on our earlier development of robust CRISPRi in mycobacteria (Rock et al., 2017) and leverages the unique PAM tuning capabilities for Sth1Cas9 relative to SpyCas9. Although it is possible to tune target knockdown by varying ATc concentrations, this approach is noisy at the single-cell level, and knockdown is dependent on target promoter strength (Vigouroux et al., 2018). Although we chose to tune by varying the sgRNA targeting sequence length, it will be important to test the use of mismatched sgRNAs, as implemented recently with SpydCas9 in *E. coli* an *B. subtilis* (Hawkins et al., 2020; Mathis et al., 2021). Prior methods to titrate gene expression in mycobacteria involved use of regulated promoters or regulated proteolysis of targeted genes (Ehrt et al., 2005; Johnson et al., 2019). Although these methods can be effective, they involve non-endogenous expression levels (regulated promoters) or the potential for perturbed function as a result of protein tagging (regulated proteolysis). Moreover, both methods are low throughput compared with genome-scale CRISPRi. Our approach further builds on the concept of gene vulnerability introduced by seminal studies in diverse organisms (Barry et al., 2009; Barve et al., 2010; Keren et al., 2016; Tonge, 2018; Wei et al., 2011).

The fact that the targets of the two most potent first-line TB drugs rank in the upper vulnerability quartile lends credence to the validity of vulnerability estimates to nominate valuable therapeutic targets. Further, the fact that drug targets like *coaA* and *def* rank in the lower quartile of vulnerability estimates may provide an explanation for why drug discovery efforts directed to these targets failed (Barry et al., 2009). The failures of target-based drug discovery are typically ascribed to compound liabilities (Payne et al., 2007); e.g., the inability to cross the bacterial envelope, efflux, and xenobiotic metabolism. We propose that target qualities may be equally important. Small-molecule inhibition of an invulnerable target is difficult; to be effective, inhibitors must reach very high levels of target engagement, likely with long residence times, to maintain sufficient target inhibition during drug dosing intervals (Tonge, 2018). Even then, sustained inhibition of an invulnerable target may not produce a fitness cost as severe as for a more vulnerable target like *rpoB*.

Our results provide a roadmap to reinvigorate target-based drug discovery in TB; all else being equal (e.g., druggability), prioritize vulnerable targets and de-prioritize invulnerable targets for drug screening. Increasing the success rate of target-based drug discovery would be particularly impactful, given the failures of this platform to robustly identify new clinical antibacterial leads (Payne et al., 2007). Care should be taken to ensure vulnerability conservation across Mtb clinical isolates because differential vulnerabilities can lead to differential drug susceptibilities. Numerous targets that are highly vulnerable in Mtb have yet to be pursued, including targets significantly more vulnerable than the current first-line TB therapies and in underexplored processes such as protein folding (*groES* and *groEL2*) and secretion (*secYE*), metabolism (*nadD*, *dxs1*, *aroF*, and *purB*), chromosome replication (*dnaE1* and *dnaA*), and cell division (*ftsZ*). Noteworthy are the tRNA synthetases, a gene class that is universally vulnerable in Mtb under the tested conditions. tRNA synthetases may present opportunities for multi-targeting of conserved active sites with a single compound to reduce resistance rates, similar to β-lactam antibiotics and penicillin-binding proteins. Focusing drug development efforts on highly vulnerable genes may have multiple benefits, including lower rates of antimicrobial resistance, given the higher levels of purifying selection and evolutionary constraints of vulnerable genes.

Why would a bacterial cell express essential gene products at levels close to those needed for optimal fitness (i.e., vulnerable genes) or well above those needed for optimal fitness (i.e., invulnerable genes)? Drawing parallels with haploinsufficiency, vulnerable genes may be dosage stabilized so that under- or overexpression reduces cellular fitness (Morrill and Amon, 2019). In this regard, it is intriguing that the most vulnerable processes are not necessarily the most highly expressed. There are numerous potential reasons to explain gene invulnerability. Higher-than-required levels of gene products could (1) impart robustness to stochastic changes in gene expression; (2) enable a rapid cellular response to environmental changes to alter pathway flux faster than upregulating gene expression; (3) represent partial functional redundancy; (4) reflect moonlighting, where a single protein performs multiple functions and elevated protein levels are required to perform all functions; and (5) reflect target levels required under growth conditions not modeled in axenic culture. Last, invulnerability could be a result of negative feedback (Vigouroux et al., 2018).

We develop and apply a method to quantify target vulnerability at genome scale in mycobacteria. Our results challenge the persistent view of gene essentiality as a binary trait, instead describing essentiality as a continuous variable. These data are being used to prioritize targets for target-based drug discovery with the goal of demonstrating *in vivo* efficacy with a small molecule inhibiting a specific mycobacterial target. It is well appreciated that gene essentiality is influenced by genetic background and growth conditions (Mathis et al., 2021). This work sets the stage for expanded vulnerability studies in different Mtb clinical strains and growth environments, including *in vivo* infection models. Our approach is readily generalizable to other bacterial pathogens, and the concepts are applicable to target ranking in other diseases, such as malaria and cancer (Behan et al., 2019; Stanway et al., 2019). To ensure wide access to this resource, all vulnerability data and visualizations are publicly available through an interactive database (https://pebble.rockefeller.edu), and CRISPRi plasmids and libraries are available through Addgene.

### Limitations of the study

There are four main limitations of our approach as it relates to drug discovery (Rock, 2019). First, CRISPRi results in a polar effect—any operonic gene downstream of the dCas9 binding site may be silenced in addition to the targeted gene (Qi et al., 2013). The facts that genes in the same operon frequently perform similar functions and many operons contain internal promoters driving transcription of downstream genes (Mejía-Almonte et al., 2020) partially mitigates this problem. Second, transcriptional interference mimics the effects of a noncompetitive inhibitor, whereas small molecules can have a wider variety of biochemical effects, from antagonism to agonism. Thus, target invulnerability should not be equated with un-druggability. Third, depletion of a target is not the same as inhibition of its functional activity by a small molecule (Knight and Shokat, 2007). For example, although a small molecule may selectively inhibit the enzymatic but not scaffolding function of an enzyme, CRISPRi will necessarily inhibit both. Fourth, invulnerability could be a result of negative feedback and reduced CRISPRi efficacy (Donati et al., 2021; Rousset et al., 2018; Vigouroux et al., 2018). We expect that, in most cases, feedback will negatively affect CRISPRi and small-molecule efficacy. Moreover, even if negative feedback were to produce a false negative vulnerability call (i.e., a gene that appears invulnerable to CRISPRi inhibition but vulnerable to small-molecule inhibition), this is much less detrimental to drug discovery than a false positive vulnerability call.

## STAR★Methods

### Key resources table

**Table:** 

REAGENT or RESOURCE	SOURCE	IDENTIFIER
**Bacterial and virus strains**		

*M. tuberculosis* H37Rv	Christopher Sassetti (UMass Worcester)	N/A
*M. tuberculosis* HN878	Clifton Barry III (NIH)	N/A
*M. smegmatis* mcˆ2 155	Sarah Fortune (Harvard)	N/A
MegaX DH10B T1R Electrocomp Cells	Invitrogen	Cat# C640003

**Chemicals, peptides, and recombinant proteins**		

Anhydrotetracycline hydrochloride	Abcam	Cat# ab145350
Q203	Pethe et al., 2013	IAP6; CAS: 1334719-95-7
ND-10885	Clifton Barry III (NIH)	N/A
Novobiocin	Sigma-Aldrich	Cat# N1628-1G
SPR719	MedChem Express	Cat# HY-12930

**Critical commercial assays**		

Renilla Luciferase Assay	Promega	Cat# E2820
SuperScript IV First-Strand Synthesis System	Thermo Fisher Scientific	Cat# 18-091-050
NextSeq 500/550 High Output Kit v2.5 (75 Cycles)	Illumina	Cat# 20024906
HiSeq 3000/4000 SBS Kit (50 cycles)	Illumina	Cat# FC-410-1001
NovaSeq 6000 S2 kit	Illumina	Cat# 20028316

**Deposited data**		

Raw sequencing data	This paper	SRA: PRJNA700384
Raw mass spectrometry data	This paper	DOI: 10.5281/zenodo.4754341

**Oligonucleotides**		

sgRNAs for CRISPRi, see Table S1	This paper	N/A
NGS primers for library amplification and sequencing, see Table S1	This paper	N/A
qRT-PCR primers, see Table S1	This paper	N/A
sgRNA oligo arrays to clone CRISPRi libraries RLC11 and RLC12	This paper	Addgene #163955 and #163954; Github: https://github.com/rock-lab/vulnerability_2021

**Recombinant DNA**		

Plasmid plRL1	Rock et al., 2017	Addgene #115162
CRISPRi plasmids	This paper	Addgene #163631; 163633; 163634; 163635; 166886
Renilla luciferase reporter plasmid	This paper	Addgene #163632
CRISPRi libraries RLC11 and RLC12	This paper	Addgene #163955 and #163954; Github: https://github.com/rock-lab/vulnerability_2021

**Software and algorithms**		

Vulnerability analysis pipeline	This paper	Github: https://github.com/rock-lab/vulnerability_2021/
Subread aligner (version 1.6.0)	Liao et al., 2013	http://subread.sourceforge.net/
Python (version 2.7.18)	van Rossum, 1995	https://www.python.org/
SciPy (version 1.2.2)	Virtanen et al., 2020	https://www.scipy.org/
statsmodels (version 0.10.1)	Seabold and Perktold, 2010	https://www.statsmodels.org/stable/index.html
Rstan (version 2.19.3)	Stan Development Team, 2020	https://mc-stan.org/
Stan (version 2.19.3)	Stan Development Team, 2021	https://mc-stan.org/
SpectroMine 1.0	Biognosys AG	https://biognosys.com/software/spectromine/

**Other**		

Resource website that provides gene vulnerability data for *M. tuberculosis* and *M. smegmatis*	This paper	https://pebble.rockefeller.edu/
Mass Spectrometer	Thermo Fisher Scientific	Orbitrap Q Exactive Plus
Liquid Chromatograph	Thermo Fisher Scientific	Easy-nLC 1200
Easy Spray HPLC column	Thermo Fisher Scientific	Cat# ES800A

### Resource availability

#### Lead contact

Further information and requests for resources and reagents should be directed to and will be fulfilled by the lead contact, Dr. Jeremy Rock (rock@rockefeller.edu).

#### Materials availability

Plasmids and CRISPRi libraries generated in this study have been deposited to Addgene. Plasmids: plRL2 (#163631); plRL19 (#163634); plRL58 (#166886); plRL61 (#163633); plRL117 (#163635); and plRL118 (#163632). CRISPRi libraries: RLC11 (#163955) and RLC12 (#163954).

#### Data and code availability

Raw sequencing data are deposited to the Short Read Archive (SRA) under project number SRA: PRJNA700384. Mass spectrometry data are deposited in Zenodo (DOI: 10.5281/zenodo.4754341). The vulnerability predictions for all targeted *M. tuberculosis* and *M. smegmatis* genes and associated visualization plots are available at https://pebble.rockefeller.edu. All source code and CRISPRi library FASTA files are publicly available online (Github: https://github.com/rock-lab/vulnerability_2021).

### Experimental model and subject details

#### Bacterial strains

*M. tuberculosis* strains are derivatives of H37Rv or HN878. *M. smegmatis* strains are derivatives of mc^2^155. *E. coli* strains are derivatives of DH5alpha.

Whole-genome sequencing (WGS) of our H37Rv clone identified 4 SNPs compared to the reference genome (GenBank: NC_018143.2). WGS of our HN878 clone identified 1,460 SNPs relative to GenBenk: NC_018143.2 as well as a common genomic duplication found during axenic expansion of Mtb clinical isolates (Carey et al., 2018; Figure S6A).

#### Mycobacterial cultures

*M. tuberculosis* and *M. smegmatis* were grown at 37°C in Difco Middlebrook 7H9 broth (BD #271310) or 7H10 (BD #262710) plates supplemented with 0.2% glycerol (7H9) or 0.5% glycerol (7H10), 0.05% Tween80, 1X albumin dextrose catalase (ADC) (*M. smegmatis*) or oleic acid ADC (*M. tuberculosis*). Where required, antibacterials or small molecules were used at the following concentrations: kanamycin (KAN) at 20 μg ml–1; anhydrotetracycline (ATc) at 100 ng ml–1.

#### Plasmid construction and cloning

Plasmid plRL2 (Addgene #163631; Table S1) or plRL58 (Addgene #166886; Table S1) were used for all *M. tuberculosis* CRISPRi experiments. plRL2 contains (1) the *Sth1 dcas9* allele under the control of an optimized, synthetic Tet repressor (TetR)-regulated promoter; (2) the *Sth1* sgRNA under the control of a synthetic TetR-regulated promoter; (3) a mycobacterial codon optimized Tet repressor; (4) a single-copy L5-integrating backbone (Lee et al., 1991); (5) a pBR322-derived *E. coli* replication origin; and (6) a kanamycin-selectable marker. plRL2 is identical to plasmid plJR965 (Rock et al., 2017) (Addgene plasmid #115163) with the exception that the *Sth1 dcas9* mRNA contains a modified Shine-Dalgarno motif (plRL2: ACGCGG; plJR965: AGGCGG) predicted to reduce Sth1 dCas9 translational efficiency. The motivation to lower Sth1 dCas9 expression levels was to ensure minimal fitness cost of dCas9 expression (Boyle et al., 2017; Vigouroux et al., 2018; Zhang and Voigt, 2018) and to further minimize any leaky expression of the CRISPRi system in the absence of ATc. plRL58 is identical to plRL2 with the exception that the L5 integrase was removed to increase plasmid stability in the absence of kanamycin selection. plRL58 was constructed by ligating olJR3549 (5′-GATCCACTTGCACTGCACC-3′) and olJR3550 (5′-CTAGGGTGCAGTGCAAGTG-3′) into BamHI- and AvrII-digested plRL2, thereby removing the L5 integrase promoter and the majority of the L5 integrase ORF. To integrate plRL58 into the mycobacterial chromosome, L5 integrase function is supplied *in trans* on a separate suicide vector, plRL19 (Addgene #163634; Table S1). Plasmid plRL19 contains the L5 integrase gene expressed from the strong mycobacterial optimized promoter (MOP) in a pBR322 plasmid backbone, thus plRL19 is non-replicating and non-integrating in mycobacteria.

Plasmid plRL117 (Addgene #163635) or plRL61 (Addgene #163633; Table S1) were used for all *M. smegmatis* CRISPRi experiments except those depicted in Figure S1, where plJR962 (Addgene #115162) was used. plRL117 is identical to plasmid plJR965 with the exception that the *Sth1 dcas9* mRNA contains a modified Shine-Dalgarno motif (plRL117: AGGCGC; plJR965: AGGCGG) predicted to reduce Sth1 dCas9 translational efficiency. plRL61 is identical to plRL117 with the exception that the L5 integrase was removed as in plRL58. Integration of plRL61 is achieved through co-transformation with plasmid plRL19.

The *Renilla* luciferase reporter plasmid plRL118 (Addgene #163632 is identical to the reporter plasmid described in (Rock et al., 2017)- with the exception that the *Renilla* luciferase gene was codon-optimized for use in mycobacteria and cloned downstream of a strong mycobacterial optimized promoter (P_MOP_).

### Method details

#### CRISPRi Library Design

##### M. tuberculosis

The *M. tuberculosis* CRISPRi Library (RLC12; Addgene #163954) was designed to target all possible *M. tuberculosis* ORFs and non-coding RNAs. 73 *M. tuberculosis* genes could not be targeted due to lack of Sth1 PAM recognition sequences. RLC12 is a combination of two sub-libraries:

1) RLC1 was designed primarily to target predicted *in vitro* essential genes.
2) RLC3 was designed solely to target predicted *in vitro* non-essential genes.

Gene essentiality predictions were sourced from an *M. tuberculosis* TnSeq meta-analysis (DeJesus et al., 2017).

To design RLC1 and RLC3, we first extracted all possible sgRNA targeting sequences in the H37Rv *M. tuberculosis* genome (GenBank: NC_018143.2) by identifying all 24 possible Sth1 dCas9 PAM sequences (Rock et al., 2017; Data S1). We then extracted 15-26 nucleotide sgRNA targeting sequences upstream of each PAM. Only sgRNA targeting sequences in which the 5′ transcription initiating nucleotide was an “A” or “G” were kept for further processing. This list represented all possible Sth1 sgRNAs targeting the H37Rv genome.

We applied different sgRNA selection criteria to predicted *in vitro* essential and non-essential genes. sgRNAs were selected according to the following criteria:

1) RLC1: for predicted essential genes, all 24 possible PAMs (Data S1) were selected for targeting. For predicted non-essential genes, only the strongest nine predicted PAMs (PAMscore_v1 = 1–9, corresponding to original *Renilla* knockdown results in Rock et al. (2017)) were selected for targeting. For a subset of predicted non-essential genes, all 24 possible PAMs were selected. Genes for which TnSeq essentiality predictions were not available were targeted with all 24 PAMs. RLC3 contains sgRNAs targeting additional PAMs based on PAM “strength” re-ranking based on the linear model results, such that each non-essential gene is targeted with a minimum of five sgRNAs.
2) sgRNA targeting sequence length was varied from 15 to 26 nucleotides for predicted essential genes; sgRNAs of lengths 21-26 nucleotides were chosen for predicted non-essential genes. For both gene classes, if an sgRNA for an individual targeted PAM of length ≥ 22 nucleotides was designed, no further longer length variants were included.
3) sgRNAs were chosen to target the non-template strand of ORFs and non-coding RNAs. sgRNA targeting sequence overlap with an ORF or non-coding RNA was defined by the 3′ base of the sgRNA targeting sequence.
4) sgRNA targeting sequences containing an internal BsmBI restriction site were removed.
5) sgRNA targeting sequences affected by a SNP in HN878 were excluded from the HN878 analysis.

We also designed non-targeting control sgRNAs. To design these sgRNAs, the Mtb genome was scrambled and sgRNAs extracted according to the design principles listed above. This approach matches the GC content of targeting and non-targeting sgRNAs. Potential non-targeting sgRNAs were mapped back to GenBank: NC_018143.2 using Bowtie (Langmead et al., 2009). Only sgRNAs with at least two mismatches relative to the parental genome (and at least one mismatch in the sgRNA seed region, here defined as the PAM-proximal 12 nucleotides) were selected as non-targeting sgRNAs for library construction. The non-targeting sgRNA length distribution was then controlled to match the gene-targeting sgRNAs.

In total, H37Rv RLC12 contains 96,700 unique sgRNAs: 63,867 sgRNAs targeting 624 TnSeq predicted *in vitro* essential genes; 29,609 sgRNAs targeting 3,237 TnSeq predicted *in vitro* non-essential genes; 1,566 sgRNAs targeting 191 genes of unknown TnSeq essentiality; and 1,658 non-targeting control sgRNAs (Addgene #163954). This represents 98.2% targeting coverage (4,052 of 4,125) of all *M. tuberculosis* ORFs and non-coding RNAs.

##### M. smegmatis

All possible sgRNA targeting sequences were extracted from the *M. smegmatis* genome (GenBank: NC_008596.1) as described above.

sgRNA design criteria for the *M. smegmatis* CRISPRi library are similar to the *M. tuberculosis* RLC12 library with the following exceptions:

1) Since there were no published TnSeq experiments to define essential genes in *M. smegmatis* at the time of library construction, we targeted all 24 possible PAMs for all genes.
2) The sgRNA targeting sequence length was varied from 17 to 24 nucleotides for sgRNAs targeting genes in *M. smegmatis* considered to be essential. Genes were considered to be essential if they had an essential *M. tuberculosis* homolog or were previously identified as essential (de Wet et al., 2020). For sgRNAs targeting an *M. smegmatis* gene with either no *M. tuberculosis* ortholog or a non-essential ortholog, sgRNA length was varied between 21-24 nucleotides.
3) We included 7,421 unique non-targeting negative control sgRNAs designed as described for the *M. tuberculosis* CRISPRi libraries.

The *M. smegmatis* CRISPRi library RLC11 (Addgene #163955) consists of 159,073 individual sgRNAs targeting 99.4% (6,642 of 6,679) of all *M. smegmatis* genes. It consists of 27,702 sgRNAs targeting 401 TnSeq predicted *in vitro* essential genes; 120,429 sgRNAs targeting 5,980 TnSeq predicted *in vitro* non-essential genes; 3,521 guides targeting 261 genes of unknown TnSeq essentiality; 17,213 guides in intergenic regions (not analyzed in this study) and 7,421 non-targeting control sgRNAs.

#### CRISPRi library production

sgRNA targeting sequence oligonucleotides were designed to encode:

1) The sgRNA targeting sequence (15-26 nucleotides in length).
2) 5′ and 3′ BsmBI restriction sites with compatible sticky end DNA overhangs for sgRNA ligation into the CRISPRi plasmid backbone.
3) 5′ and 3′ primer binding sites for PCR amplification.

##### M. tuberculosis (RLC12)

As described in *CRISPRi Library Design*, RLC12 is a combination of the *M. tuberculosis* CRISPRi libraries RLC1 and RLC3. Oligonucleotides were synthesized by CustomArray (92,918 oligo pool; RLC1) or Agilent Technologies (SureGuide Custom CRISPR Guide Library #G7555B#100; RLC3). See Addgene #163954 for oligonucleotide sequences.

To generate RLC1, 60 μg of plRL2 (see Plasmid construction and cloning) was digested with BsmBI (NEB #R0580) and gel purified (QIAGEN #28706). BsmBI-digested plRL2 was then further cleaned and concentrated by ethanol precipitation. Next, the pooled sgRNA oligonucleotide library was PCR amplified using NEBNext High-Fidelity 2X PCR Master Mix (NEB #M0541L). Seventy-two 50 μL PCR reactions were prepared, where each reaction contained 25 μL of PCR master mix, 0.05 pmol of the oligonucleotide library, and a final concentration of 0.5 μM of the appropriate forward and reverse primers (Fwd: 5′-GGGACGATCTGCTGTGTATAGAG-3′ + Rv: 5′-CCTGCTCCCAATGTACCCT-3′). PCR cycling conditions were: 98°C for 30 s; 14 cycles of 98°C for 10 s, 67°C for 10 s, 72°C for 15 s; 72°C for 120 s. PCR amplicons were purified using the QIAGEN MinElute PCR purification kit (QIAGEN #28004). Next, the purified amplicons were digested with FastDigest Esp3I (Thermo Scientific #FD0454), PAGE purified on a 4%–20% polyacrylamide gel (Invitrogen #XV04205PK20) and isopropanol precipitated. Twenty-four ligation reactions (T4 DNA ligase NEB #M0202M) were prepared, each with 500 ng of BsmBI-digested plRL2 and 5 ng of Esp3I-digested sgRNA targeting sequences, representing a 1:4 molar ratio of vector:insert. Ligations were incubated overnight at 16°C. Following ligation, the products were purified and concentrated using a DNA Clean & Concentrator-25 kit (Zymo #D4034) and spot dialyzed (Millipore #VSWP02500).

The RLC3 cloning approach was similar to RLC1 with the following exceptions:

1) The pooled sgRNA oligonucleotide library was amplified using Q5 High-Fidelity Master Mix (NEB #M0492L). A total of eight 50 μL PCR reactions were performed. Each reaction contained 25 μL of Q5 master mix, 0.05 pmol of the oligonucleotide library and a final concentration of 0.5 μM of the appropriate forward and reverse primers (Fwd: 5′-GGGACGATCTGCTGTGTATAGAG-3′ + Rv: 5′-CCTGCTCCCAATGTACCCT-3′). PCR cycling conditions were: 98°C for 30 s; 10 cycles of 98°C for 10 s, 64°C for 10 s, 72°C for 15 s; 72°C for 120 s. PCR amplicons were purified using the QIAGEN MinElute PCR purification kit (QIAGEN #28004).
2) sgRNA targeting sequences were cloned into plRL2 by Golden Gate cloning (Fromme and Klingenspor, 2007). Each 20 μL Golden Gate reaction (eight reactions in total) contained 800 fmol of PCR amplicon, 80 fmol of BsmBI-digested plRL2, 20 mM DTT, 20 mM ATP, 1X FastDigest Buffer, 10 U of FastDigest Esp3I (Thermo Scientific #FD0454) and 1,000 units T4 DNA ligase (NEB #M0202M). Cycling conditions were: 50 cycles of 37°C for 5 min and 16°C for 5 minutes, followed by 55°C for 1 hour and a 4°C hold. Upon completion, 5U of Esp3I was added per 20 μL reaction and the mix incubated at 37°C for 1 hour. The Golden Gate reactions were terminated by heat killing at 80°C for 5 min.

##### M. smegmatis (RLC11)

The M. smegmatis sgRNA library was PCR amplified and cloned into plasmid plRL117 (see Plasmid construction and cloning). The RLC11 cloning approach was similar to RLC3 with the following exceptions:

1) sgRNA targeting sequence oligonucleotides were designed to encode two sgRNA targeting sequences on the same oligonucleotide. Each sgRNA targeting sequence was flanked with BsmBI restriction sites and compatible sticky end DNA overhangs. The sgRNA oligonucleotides were synthesized by CustomArray (92,918 oligo pool). See Addgene #163955 for oligonucleotide sequences.
2) To amplify the oligonucleotides, a total of forty 50 μL PCR reactions were performed. Each reaction contained 25 μL of Q5 master mix, 0.05 pmol of the library and a final concentration of 0.5 μM of the appropriate forward and reverse primers (Fwd: 5′-GGGACGATCTGCTGTGTATAGAG-3′ + Rv: 5′- CCTGCTCCCAATGTAGCCT-3′). PCR cycling conditions were: 98°C for 30 s; 11 cycles of 98°C for 10 s, 67°C for 10 s, 72°C for 15 s; 72°C for 120 s.

#### *E. coli* library transformations

All CRISPRi sgRNA libraries were transformed into MegaX DH10B T1R Electrocomp Cells (Invitrogen #C640003).

RLC1: 3 μg of the dialyzed ligation (53 μL) was added to 600 μL of MegaX cells. The mixture was supplemented with 600 μL of ice-cold glycerol. A total of 16 transformations were performed. For each transformation, 75 μL of the cells:DNA mix was transferred to a 0.1 cm electroporation cuvette (BioRad #1652089) and electroporated at 2,000 V, 200 ohms, 25 μF. The transformations were recovered in a total of 20 mL SOC medium with shaking for 1.5 hours at 37°C. Following recovery, bacteria were spread (650 μL per plate) on prewarmed LB Miller agar supplemented with kanamycin (50 μg/mL) in Corning Bioassay dishes (Sigma #CLS431111-16EA). The plates were incubated at 37°C for 18 h. Following outgrowth, transformants were harvested by scraping. The CRISPRi plasmid library was then isolated using a QIAGEN Plasmid Giga Kit (QIAGEN #12191). Finally, the quality of the sgRNA library was confirmed by deep sequencing (see Genomic DNA extraction and library preparation for Illumina sequencing).

RLC3 and RLC11 were transformed similar to the method described for RLC1 with the following exceptions:

1) Nine transformations were performed for RLC3. 1.5 μg of the dialyzed Golden Gate reaction (21.6 μL) and 300 μL MegaX cells were used. The recovered cells were plated on 15 Corning Bioassay dishes.
2) Forty-five transformations were performed for RLC11. 7.5 μg of the dialyzed Golden Gate reaction (107 μL) and 1,500 μL MegaX cells were used. The recovered cells were plated on 75 Corning Bioassay dishes.

#### CRISPRi library transformation

Validated CRISPRi libraries (see Genomic DNA extraction and library preparation for Illumina sequencing) were electroporated into mycobacteria as described in Murphy et al. (2015).

##### M. tuberculosis

###### H37Rv

Sixty transformations were performed to generate *M. tuberculosis* RLC1 libraries. For each transformation, 1 μg of RLC12 plasmid DNA was added to 200 μL electrocompetent cells (∼4 × 10^9^ cells per transformation). The cells:DNA mix was transferred to a 2 mm electroporation cuvette (Bio-Rad #1652082) and electroporated at 2500 kV, 700 ohms, and 25 μF. Each transformation was recovered in 5 mL 7H9 media supplemented with OADC, glycerol and Tween80 (300 mL total) for 16-24 hours. The recovered cells were harvested at 4,000 rpm for 10 minutes, resuspended in 700 μL remaining media per transformation and plated on 7H10 agar supplemented with kanamycin (see Bacterial cultures) in Corning Bioassay dishes (Sigma #CLS431111-16EA). Transformation efficiency was estimated from library titering and indicated > 80X average sgRNA coverage of RLC1 was achieved in *M. tuberculosis*.

After 21 days of outgrowth on plates, transformants were scraped and pooled. Scraped cells were homogenized by two dissociation cycles on a gentleMACS Octo Dissociator (Miltenyi Biotec #130095937) using the RNA_01 program and 30 gentleMACS M tubes (Miltenyi Biotec #130093236). The library was further declumped by passaging 10 individual *M. tuberculosis* library aliquots in 10 mL of 7H9 supplemented kanamycin (see Mycobacterial cultures) in T-25 flasks (Falcon # 08-772-1F) for 15 generations. Final *M. tuberculosis* RLC1 library stocks were obtained after pooling the cultures and passing them through a 10 μm cell strainer (Pluriselect #SKU 43-50010-03). Genomic DNA was extracted from the final *M. tuberculosis* RLC1 library stock and library quality was validated by deep sequencing (see Genomic DNA extraction and library preparation for Illumina sequencing).

The *M. tuberculosis* RLC3 library was generated similar to RLC1, with the exception that only eight *M. tuberculosis* transformations were performed due to the smaller library size. Library titering indicated ∼130X average sgRNA coverage of RLC3 was achieved in *M. tuberculosis*.

To generate RLC12, five 1 mL aliquots of the final homogenized RLC1 library and one aliquot of the final homogenized RLC3 library were thawed. Each aliquot was inoculated into 24 mL 7H9 media supplemented with kanamycin in a T-75 flask (starting OD_600_∼0.04; which represents ∼3,000X coverage of RLC1 and ∼15,000X coverage of RLC3). The cultures were expanded to OD_600_∼2, passed through a 10 μm cell strainer (pluriSelect #43-50010-03) to obtain a single cell suspension and pooled in a 1:0.18 ratio corresponding to relative library size. The OD_600_ of the mixture was adjusted to 1.0 and aliquots were frozen at –80°C. Genomic DNA was extracted from the pooled library and library quality was validated by deep sequencing (see Genomic DNA extraction and library preparation for Illumina sequencing).

###### HN878

The HN878 *M. tuberculosis* RLC12 library was generated similar to the H37Rv RLC12 library with the following exceptions: 1) Due to the anticipated lower transformation efficiency in HN878, sixty-eight transformations were performed for HN878 with RLC1 library plasmid; the final coverage was > 30X. 2) To generate RLC3, 12 HN878 transformations were performed; the final coverage was > 1,500X. 3) To generate the final HN878 RLC12 library, three 1 mL aliquots of the final homogenized HN878 RLC1 library and one aliquot of the final homogenized HN878 RLC3 library were thawed and mixed similar to H37Rv.

##### M. smegmatis

The *M. smegmatis* RLC11 library was generated similar to RLC1 with the following exceptions:

1) 100 μg of RLC11 plasmid library was added to 10 mL of *M. smegmatis* electrocompetent cells (3 × 10^11^ cells). This pool was transformed in 100 individual transformations and recovered in a total of 300 mL of 7H9 media supplemented (see Mycobacterial cultures) for 5 hours.
2) Recovered cells were pelleted at 4,000 x g for 10 min and resuspended in 50 mL remaining media. The cells were plated on 7H10 agar supplemented with kanamycin (see Mycobacterial cultures) in Corning Bioassay dishes (Sigma #CLS431111-16EA) and incubated at 37°C for 3 days.
3) Library titering showed > 1,600X average sgRNA coverage of RLC11 was achieved in *M. smegmatis.*

#### Pooled CRISPRi screen

##### M. tuberculosis

Pooled CRISPRi screens were performed in vented tissue culture flasks (T-75; Falcon #353136). Twenty mL cultures were grown in 7H9 media supplemented with kanamycin (see Mycobacterial cultures) and maintained at 37°C, 5% CO2 in a humidified incubator.

The screen was initiated by thawing four 1 mL aliquots of the *M. tuberculosis* (H37Rv or HN878) CRISPRi library (RLC12) and inoculating each aliquot into 19 mL 7H9 media supplemented with kanamycin in a T-75 flask (starting OD_600_∼0.06). The cultures were expanded to OD_600_ = 1.5, pooled and passed through a 10 μm cell strainer (pluriSelect #43-50010-03) to obtain a single cell suspension. The single cell suspension (flow-though) was used to set up six “generation 0” cultures: three replicate cultures with ATc (+ATc) and three replicate control cultures without ATc (–ATc). From each generation 0 culture, we harvested 10 OD_600_ units of bacteria (∼3x10^9^ bacteria; ∼30,000X coverage of the CRISPRi library) for genomic DNA extraction. The remaining culture volume was used to initiate the pooled CRISPRi fitness screen. Cultures were periodically passaged in pre-warmed media in order to maintain log phase growth. At generation 2.5, 5, and 7.5, cultures were back-diluted 1:6 (to a starting OD_600_ = 0.2) and cultivated for approximately 2.5 doublings. At generation 10, 15, 20, and 25, cultures were back-diluted 1:24 (to a starting OD_600_ = 0.05) and expanded for 5 generations before reaching late-log phase. ATc was replenished at every passage. By keeping the OD_600_ of the 20 mL cultures ≥ 0.05, we guaranteed sufficient coverage of the library (3,000X) at all times. At set time points (approximately 2.5; 5; 7.5; 10; 15; 20; 25 and 30 generations), we harvested bacterial pellets (10 OD_600_ units) to extract genomic DNA.

##### M. smegmatis

The *M. smegmatis* pooled CRISPRi screen was performed similar to the *M. tuberculosis* screens described above with two primary differences: 1) the growth media contains ADC (see Mycobacterial cultures); and 2) cultures were grown shaking at 120 rpm in vented 125 mL Erlenmeyer flasks (Thermofisher #4115-0125).

#### Genomic DNA extraction and library preparation for Illumina sequencing

Genomic DNA was isolated from bacterial pellets using the CTAB-lysozyme method described by Larsen et al. (2007). Genomic DNA concentration was quantified using the DeNovix dsDNA high sensitivity assay (KIT-DSDNA-HIGH-2; DS-11 Series Spectrophotometer / Fluorometer). Next, the sgRNA-encoding region was amplified from 500 ng genomic DNA using NEBNext Ultra II Q5 master Mix (NEB #M0544L). PCR cycling conditions were: 98°C for 45 s; 17 cycles of 98°C for 10 s, 64°C for 30 s, 65°C for 20 s; 65°C for 5 min. Each PCR reaction contained a pool of forward primers (0.5 μM final concentration) and a unique indexed reverse primer (0.5 μM) (Table S1). Forward primers contain a P5 flow cell attachment sequence, a standard Read1 Illumina sequencing primer binding site, and custom stagger sequences to ensure base diversity during Illumina sequencing. Reverse primers contain a P7 flow cell attachment sequence, a standard Read2 Illumina sequencing primer binding site, and unique barcodes to allow for sample pooling during deep sequencing.

Following PCR amplification, each ∼230 bp amplicon was purified using AMPure XP beads (Beckman–Coulter #A63882) using one-sided selection (1.2x). Bead-purified amplicons were further purified on a Pippin HT 2% agarose gel cassette (target range 180-250; Sage Science #HTC2010) to remove primer carry-over and genomic DNA. Eluted amplicons were quantified with a Qubit 2.0 Fluorometer (Invitrogen), and amplicon size and purity were quality controlled by visualization on an Agilent 2100 Bioanalyzer (high sensitivity chip; Agilent Technologies #5067-4626). Next, individual PCR amplicons were multiplexed into 10 nM pools and sequenced on an Illumina sequencer according to the manufacturer’s instructions (2.5%–5% PhiX spike-in; PhiX Sequencing Control v3; Illumina # FC-110-3001). Samples were run on the Illumina NextSeq 500, HiSeq 4000, or NovaSeq 6000 platform (Single-Read 1x85 cycles and six i7 index cycles). Samples were sequenced to achieve a target sgRNA median count depth of approximately 750-1,000 per sample in *M. tuberculosis* and *M. smegmatis*.

#### Generation of individual CRISPRi strains

Individual CRISPRi plasmids were cloned as previously described (Rock et al., 2017) with minor modifications (see (Wong and Rock, 2021)). Briefly, the CRISPRi plasmid backbone (see Plasmid construction and cloning) was digested with BsmBI-v2 (NEB #R0739L) and gel purified. sgRNAs were designed to target the non-template strand of the target gene ORF. For each individual sgRNA, two complementary oligonucleotides with appropriate sticky end overhangs were annealed and ligated (T4 ligase NEB # M0202M) into the BsmBI-v2 digested plasmid backbone. Successful cloning was confirmed by Sanger sequencing. A list of sgRNA targeting sequences and plasmids used for constructing individual CRISPRi strains can be found in Table S1.

Individual CRISPRi plasmids were then electroporated into mycobacteria, recovered, and plated as described under CRISPRi library transformation into mycobacteria. For each transformation, 100 ng plasmid DNA and 100 μL of electrocompetent mycobacteria were used. Where necessary, 100ng of plasmid plRL19 was also added (see Plasmid construction and cloning).

#### Renilla luciferase assay

Twenty-nine sgRNAs spanning a range of predicted strengths (0.018-0.973; based on the H37Rv linear model coefficients) targeting the *Renilla* gene (plRL118) were designed. RNAfold (Hofacker, 2003) was used to predict secondary structure within the sgRNA targeting sequence; sgRNAs with a predicted hairpin within the sgRNA targeting sequence with a stability < –8 kcal/mol were excluded. We also designed an sgRNA that cannot target the *M. smegmatis* genome. This non-targeting sgRNA has at least 7 mismatches or indels relative to any potential target sequence in the *M. smegmatis* genome. Sequences of all sgRNAs can be found in Table S1.

*Renilla*-targeting sgRNAs were cloned into plRL117. Upon sequence confirmation, the plasmids were transformed into electrocompetent *M. smegmatis* cells containing plRL118. Cultures were grown to log phase in the absence of ATc and then diluted back to OD_600_ = 0.013 with or without 100 ng/ml ATc. After 24 hours of outgrowth, 0.8 OD_600_ units of cells was harvested by centrifugation and processed for *Renilla* luciferase assay as per manufacturer’s instructions (Promega Renilla Luciferase Assay system; #E2820). Luciferase activity was quantified in 96-well white plates (Costar; #3362) using a Spark multimode microplate reader (Tecan). Error bars represent standard error of three technical replicates.

#### ATc titration

The ATc titration assay was performed in 7H9 supplemented with 0.2% Glycerol, 0.05% Tween 80 and 10% OADC. Cultures (n = 6) were inoculated in 384-well plates (VWR; #82051-282) at OD_600_ = 0.003 and exposed to 5- or 2-fold dilutions of ATc (Abcam; ab145350) in DMSO printed with an HP D300e Digital Dispenser (Tecan). After 40 hours of incubation at 37°C with shaking, OD_600_ was measured in a plate reader (Tecan) and percent growth calculated relative to each strains growth in the absence of ATc.

#### Preparation of cell lysates for MS and qRT-PCR

*M. smegmatis* genes for validation by qRT-PCR and mass spectrometry were chosen by the following criteria:

1) The target gene should have a homolog of similar vulnerability in *M. tuberculosis*: *ms0317* + *rv0227c*, *mmpL3* (*ms0250* + *rv0206c*); *glyS* (*ms4485* + *rv2357c*); *gatB* (*ms2367* + *rv3009c*); *ms2782* + *rv2676c*; *ms4700* + *rv2477c*.
2) The target gene should not be directly upstream of an essential gene to avoid the contribution of polar effect to the growth defect.
3) The target gene should be accurately quantifiable by our label-free mass spectrometry approach (see Label-free mass spectrometry) by testing that 5 peptides are quantified with a coefficient of variation ≤ 10%.
4) RLC12 should contain at least one strong sgRNA (predicted strength > 0.90) and one hypomorphic sgRNA that has a depletion slope (β_e_) between –0.2 and –0.3 and a lag time (γ) of 4-9 generations. This β_e_ - γ matching of all tested hypomorphic sgRNAs guaranteed that the fitness cost imposed by the hypomorphic sgRNAs was similar between all targets and reached steady state by 15 generations +ATc (time of harvest).
5) sgRNA targeting sequences that contained a stable hairpin (< –8 kcal/mol) within the targeting sequence were excluded.

All samples were prepared in biological triplicate. Bacterial cultures were grown to log phase and were then diluted back to OD_60_ 0.05 with or without 100 ng/ml ATc. Back dilution was repeated twice guaranteeing cells were harvested at steady state growth (∼15 generations +ATc).

#### qRT-PCR

mRNA extraction was performed as previously described (Rock et al., 2017). Briefly, for *M. tuberculosis* cultures, 2.5 OD_600_ unit of bacteria (∼7.5x10^8^ cells) were added to 3 mL of guanidium thiocyanate (GTC) 5M buffer (Goldbio #G-210-1) and were pelleted by centrifugation. For *M. smegmatis* cultures, 1 OD_600_ unit of bacteria from uninduced and ATc-treated cultures were directly pelleted by in the absence of GTC. Next, the pellets were resuspended in 1 mL TRIzol (Thermo Fisher Scientific; #15596026) and lysed by bead beating (MP Biomedicals; #116911050). 0.2 mL chloroform was added to each sample, samples were centrifuged to separate phases, and the aqueous phase was purified by Direct-zol RNA miniprep (Zymo Research; # R2052). Residual genomic DNA was removed by TURBO DNase treatment (Invitrogen Ambion; # AM2238). After RNA cleanup and concentration (Zymo Research; #R1017), 3 μg of RNA per sample was reverse transcribed into cDNA with random hexamers (Thermo Fisher Scientific; #18-091-050). RNA was removed by alkaline hydrolysis and cDNA was purified with QIAGEN PCR clean-up columns (#28106). Next, knockdown of the CRISPRi targets was quantified by SYBR green dye-based quantitative real-time PCR (Applied Biosystems; #4309155) on a Quantstudio system 5 (Thermofisher Scientific; #A28140) using gene-specific qPCR primers (5 μM). For strains in Figure S3J, knockdown was normalized to sigA (*ms2758*) and quantified by the ΔΔCt algorithm. Strains in Figure S6H were normalized to their –ATc counterpart and the non-targeting control sgRNA. All gene-specific qPCR primers were designed using the PrimerQuest tool from IDT (http://www.idtdna.com/pages/tools/primerquest?returnurl=%2FPrimerQuest%2FHome%2FIndex) and then validated for efficiency and linear range of amplification using standard qPCR approaches. Specificity was confirmed for each validated qPCR primer pair by melt curve analysis.

#### Label-free mass spectrometry

Forty OD_600_ units of bacteria from cultures grown in the presence or absence of ATc were pelleted by centrifugation at 3500 x g for 10 min at 4°C and washed once in 10% glycerol. The cell pellet was resuspended in 0.3 mL sterile PBS containing cOmplete EDTA-free protease inhibitor cocktail (Roche; #04693159001). Then, 100 μL of 0.1 mm sterile silica beads washed with MilliQ water (BioSpec; #11079101Z) were added and the samples were bead-beat four times for 30 s each time at 10,000 rpm at 4°C (Bertin instruments; Precellys). The samples were then incubated with SDS (final concentration = 3.75%) at 80°C for 30 minutes, diluted 1:1 in a reducing buffer (20% glycerol, 50 mM TCEP, 0.5 mM EDTA, 0.05% (w/v) bromophenol blue) and incubated at 72°C for 10 minutes. Finally, the samples were treated for 30 minutes at room temperature in the dark with a final concentration of 30 mM iodoacetamide. The cell lysates were then collected by centrifugation and filtered through 0.2 μm filter spin columns (Millipore Sigma; #UFC30GV0S). Approximately 5 μg of protein lysate per sample was loaded on NuPAGE 10% Bis-Tris polyacrylamide gels (ThermoFisher Scientific, #NP0301BOX) in the presence of antioxidant (ThermoFisher Scientific, #NP0005) and run for 2.5 min at 120kV to produce a single unseparated band containing all the proteins in the sample (which we term a gel plug). The gels were Coomassie stained (Coomassie solution: Coomassie Brilliant Blue R250 (ThermoFisher Scientific; #20278) 0.5%, methanol 45%, acetic acid 10%) for 5 minutes, and destained overnight (destain solution: 16% methanol, 10% acetic acid).

The gel plug was then excised, diced into small pieces with a sharp blade, destained using 400 μL of 50mM ammonium bicarbonate in 50% Methanol: 50% H_2_O four times to efficiently remove the Coomassie blue, and digested with trypsin. The resulting peptides were extracted from the gel sequentially first using 200 μL ACN and then using 40 μL 10% Formic acid in 50%ACN:50%H2O followed by adding 200 μL 0.2%TFA in 50%ACN:50%H2O. After repeated centrifugation in a micro-centrifuge to remove gel debris followed by depletion of ACN in a SpeedVac vacuum concentrator (Thermo Fisher Scientific), the concentrated peptide solution was divided into two parts and the peptides were bound to C18 Ziptips (Millipore Sigma) (or alternatively C18 StageTips (produced in-house although available commercially from Thermo Scientific)). Peptides eluted from the Ziptip (or StageTip) were loaded onto an Easy-Spray reversed phase HPLC column (ES800A, Thermo Fisher Scientific) and analyzed by liquid chromatography–mass spectrometry (LCMS) using an Orbitrap Q Exactive Plus mass spectrometer (Thermo Fisher Scientific) coupled with an Easy-nLC system (Thermo Fisher Scientific). For the set of samples from knockdown strains and from their corresponding controls, the instrument method included targeted fragmentations on chosen peptides from the knockdown proteins.

SpectroMine (Biognosys AG) software was used for label-free quantitation (LFQ) analyses, where the area under the monoisotopic peak of a given peptide species in the Extracted Ion Chromatogram is designated the peptide LFQ and the protein LFQ is the sum of the top n peptide LFQs (n = 5, when the number of identified peptides > 5) for each protein. The peptide and protein LFQ outputs from SpectroMine were further analyzed using Microsoft Excel. To compare LFQs across samples within a given batch of samples (where a batch includes biological replicates and technical replicates of given knockdown strain(s) and knockdown corresponding controls), normalization was applied so that the sum of the normalized LFQs for 5-6 abundant proteins from each run was the same. After normalization the relative standard deviations of LFQs across samples for these 5-6 proteins were within 20%. Protein fold knockdowns (KDs) and standard deviations for the KD proteins in the hypomorphic strains versus controls were derived using the average normalized LFQs and their standard deviations (by error propagation).

#### MIC assay

H37Rv and HN878 MIC assays were performed in 7H9 supplemented with 0.2% Glycerol, 0.05% Tween 80 and 10% OADC. Cultures were inoculated in a 384-well plate (VWR #82051-282) at OD_580_ = 0.003 and exposed to √2- or 2-fold dilutions of the indicated antibacterial. At 7, 14 and 21 days of exposure OD_580_ was measured and the MIC was calculated using the Gompertz equation. The following compounds were tested: isoniazid (Sigma-Aldrich #I3377-5G), ethambutol (Sigma-Aldrich #E4630-25G), rifampicin (Sigma-Aldrich #R3501-5G); the imidazopyridine amide compounds Q203 (Pethe et al., 2013) and ND-10885 (Moraski et al., 2016), and the gyrase inhibitors novobiocin (Sigma-Aldrich #N1628-1G) and SPR719 (MedChem Express #HY-12930). Q203 and ND-10885 were a generous gift of Helena Boshoff and Clif Barry.

### Quantification and statistical analysis

#### Quantification of sgRNA depletion

To count the abundance of sgRNAs, sequencing reads were aligned to the sgRNA library sequences using *subread-align* (version 1.6.0) with default parameters (Liao et al., 2013). sgRNA library sequences included four bases of plasmid sequence upstream and downstream of the sgRNA targeting sequence, thereby enabling accurate counting of sgRNA targeting sequence length variants.

To quantify sgRNA depletion in the competitive growth experiments, mean sgRNA read counts between triplicate samples were calculated. Differences in sequencing depth between samples were normalized using the Trimmed Total Reads (TTR) method (DeJesus et al., 2017). sgRNA depletion was quantified by estimating log2 fold-change (L2FC) of the mean count in the +ATc samples relative to the mean counts in the –ATc samples. The non-targeting control sgRNAs were used in a second normalization step, which set their median L2FC to 0:

enrichment_ratio=mNT+ATcmNT−ATc

$$C_{norm}^{-ATc}=C^{-ATc}*enrichment\_ratio$$

where $m_{NT}^{+ATc}$ and $m_{NT}^{-ATc}$represent the median counts for the non-targeting sgRNAs in conditions with (+) and without (–) ATc, and $C^{-ATc}$ and $C_{norm}^{-ATc}$ represent the counts in the minus ATc condition before and after the normalization.

#### Modeling sgRNA depletion over time

sgRNA depletion in the competitive growth experiments was modeled using a piecewise linear regression model, referred to as a “two-line model.” The first line segment captures the phenotypic lag between CRISPRi induction and resulting effects on bacterial fitness and is described by the following equation:

$$Y\;=\;\alpha_{l}\;+\;\beta_{l}\text{X}$$

where Y represents the sgRNA L2FC, X is number of generations, $\alpha_{l}\;$is the sgRNA L2FC at the start of the experiment, and $\beta_{l}$ represents the slope of the lag phase. The first line is applied for X values occurring beforeγ, or the inflection point at which bacterial fitness is impacted by CRISPRi (i.e., X<γ). The second line segment captures bacterial fitness after the phenotypic inflection point (i.e., X≥γ) and is described by the following equation:

$$Y\;=\;\left(\alpha_{l}\;+\;\beta_{l}\gamma \right)+\;\beta_{e}*\left(X-\gamma \right)$$

where $\beta_{e}$ represents the rate of depletion of a given sgRNA over time. All parameters of the two-line model were fit using the L-BFGS-B optimization algorithm from the SciPy package (version 1.2.2) (Virtanen et al., 2020) for Python (version 2.7.18) (van Rossum, 1995). Full results are available in Data S1 and more details are provided in the source code (see Data and code availability).

#### Identification and validation of CRISPRi-resistant cell subpopulations

Analysis of sgRNA behavior in the competitive fitness experiments revealed some sgRNAs targeting predicted *in vitro* essential genes that exhibited unexpected behaviors. These “flatliner” sgRNAs initially depleted over time but at variable time points in the experiment (depending on the sgRNA analyzed) stopped depleting and remained at a constant relative abundance after this point, resulting in a “flatline” of the sgRNA L2FC values. The behavior of these sgRNAs indicated that these cell sub-populations are ATc-resistant. We hypothesized that at least some of these flatliners arose due to stochastic inactivation of the CRISPRi machinery during library cloning and/or transformation. To test this hypothesis, we cloned strong sgRNAs targeting four essential *M. smegmatis* genes: *gyrB* (*ms0005*), *dnaE1* (*ms3178*), *mmpL3* (*ms0250)* and *pptT (ms2648)* into plasmid plJR962 (Rock et al., 2017). We then transformed 100 ng of each plasmid into electrocompetent *M. smegmatis* and plated recovered transformants on 7H10 plates supplemented with or without ATc. Titering indicated that ∼1:1,000 transformants were ATc-resistant. Eight ATc-resistant colonies from each transformation were further single colony purified and genomic DNA was isolated. To determine the cause of CRISPRi inactivation, the entire dCas9 and sgRNA region of each plasmid was PCR amplified and submitted for Sanger sequencing. These results demonstrated that a sub-population of transformants become CRISPRi-resistant as a result of deletions within the CRISPRi machinery, most commonly variable-length deletions within Sth1 dCas9. These results are consistent with the hypothesis that at least some fraction of the flatliner sgRNAs observed in the competitive fitness experiments are a result of a CRISPRi-resistant subpopulation of transformants.

To prevent flatliner behavior from influencing vulnerability estimates, we identified flatliner sgRNAs and ignored L2FC data points after the transition point to ATc-resistance. To identify flatliners, the two-line model described above was extended to include a third line segment. This third line captures the transition of sgRNA depletion to a phase of sgRNA L2FC that no longer depletes or enriches (a “recovery” or “flatline” phase) and is represented by the following equation:

$$Y\;=\;\left(\alpha_{l}\;+\;\beta_{l}\gamma \right)+\;\beta_{e}*\left(\rho -\gamma \right)+\beta_{r}*\left(X-\rho \right)$$

where $\beta_{r}$ is the slope of the third segment, representing the rate at which the previous trend of depletion has changed (or “flatlined”) and ρ represents the transition point from depletion to flatline. The third line is applied for X values occurring after ρ (i.e., X≥ρ). This expanded model was fit to all sgRNAs in the CRISPRi libraries using the L-BFGS-B optimization algorithm as described above. After obtaining the parameter fits, sgRNAs were defined as flatliners if they met the following criteria:

• The “flatline” occurred at least four generations prior to the end of the experiment (i.e., $x^{last}-\rho >4$), and
• The L2FC at the point where the sgRNA transitions to the recovery phase (ρ) was less than –2 (i.e., Y(ρ)<−2)

and either this criterion:

• The difference in L2FC at the last time point $\left(x^{last}\right)$ and at the point the sgRNA transitions to the depletion phase (γ) is greater than –2 (i.e., $Y\left(x^{last}\right)-\gamma >-2$)

or both these criteria:

• The rate of depletion $\left(\beta_{e}\right)$ was less than –0.1 (i.e.,$\beta_{e}\;<-0.1$), and
• The slope of the recovery phase $\left(\beta_{r}\right)$ was greater than –0.05 (i.e., $\beta_{r}\;>-0.05$)

where $x^{last}\;$represents the last time point for which there is data for this guide in the time-course experiment. For any sgRNAs that met these conditions, only data points before the start of the recovery phase (ρ) were utilized in subsequent analyses.

#### Prediction of sgRNA strength

sgRNA strength was predicted using ordinary least-squares (OLS) regression. The rate of depletion (fitness cost) imposed by each sgRNA, $\beta_{e}$, identified by the two-line model described in section Modeling sgRNA depletion over time, was predicted as a function of the PAM sequence, the sgRNA targeting sequence length, and the GC-content of the sgRNA. Additional features including the presence of homopolymers within the sgRNA targeting sequence and the relative distance of the sgRNA targeting site to the start codon of the gene ORF were also analyzed but found to not significantly contribute to sgRNA strength. As only sgRNAs targeting essential genes are expected to deplete, only sgRNAs targeting genes predicted to be Essential by TnSeq (DeJesus et al., 2017) were utilized for this step. The OLS function of the *statsmodels* package (version 0.10.1) (Seabold and Perktold, 2010) for Python (version 2.7.18) was used to fit the $\beta_{e}$ for each sgRNA targeting an essential gene as a function of the three factors:

$$\beta_{e}\;∼\;PAM+Length+GC\;$$

The coefficients estimated by OLS regression were converted to a strength value ranging from 0 to 1 by normalizing the sum of the three coefficients (or “weights” associated with targeted PAM, sgRNA targeting sequence length, and sgRNA GC content), such that any combination of features with a positive coefficient (i.e., weakest depletion) equaled a strength of 0 and the strongest combination of three features equaled 1 (Data S1).

#### Gene-level depletion

To summarize the depletion of sgRNAs at the gene-level, the L2FC values of the individual gene-targeting sgRNAs were grouped into two distinct clusters using k-means clustering: one cluster representing guides that deplete and the other cluster representing guides that do not deplete (i.e., L2FC near 0). To ensure that clusters were large enough to represent distinct groups, both clusters had to contain at least 5 sgRNAs. Gene-level L2FC was taken as the most negative mean L2FC of the two clusters (if both clusters had enough guides). If both clusters did not contain at least 5 sgRNAs, gene-level L2FC was summarized as the mean L2FC of all guides.

#### Gene-level essentiality predictions

CRISPRi gene essentiality predictions were made using a modified version of the resampling approach previously utilized for TnSeq gene essentiality predictions in *M. tuberculosis* (DeJesus et al., 2017). Briefly, read-counts at 24.3 generations were compared ± ATc. Read-counts were normalized in two steps as described in *Quantification of sgRNA depletion*; first to account for sequencing depth (using TTR), and then to make use of the control sgRNAs. For each gene, normalized counts were permuted across the +ATc and –ATc conditions at 24.3 generations for a total of 20,000 iterations. While permutation tests typically look for differences in mean counts, the presence of sgRNAs of different strengths can disproportionately affect the mean of a given gene (e.g., a gene targeted with many weak and few strong sgRNAs). This made differences at lower percentiles the more relevant test-statistic, as it would be more sensitive to the presence of just a few strong guides. Thus, at each iteration, i, the difference in the 20th percentile between the counts was estimated:

$$\Delta P_{i}^{20\%}=P^{20\%}\left(C_{norm}^{B,g}\right)-P^{20\%}\left(C_{norm}^{A}\right)$$

where $P^{20\%}$is the percentile function, $C_{norm}^{A,g}$ represents normalized counts for gene g at a given conditionA. The 20,000 instances of the test-statistic estimated after all iterations represented the distribution of the test-statistic under the null-hypothesis. A p-value was estimated by comparing the observed value of the test-statistic to the distribution of the null-hypothesis. p-values were adjusted for multiple comparisons using the Benjamini-Hochberg procedure (Benjamini and Hochberg, 1995). A p-value threshold of $p_{adj}<0.01$was used to assess statistical significance.

For each gene, a summary L2FC was estimated to assess the biological significance of the effect size. L2FC was summarized as the median value of the strongest 10 sgRNAs (i.e., sgRNAs with the smallest L2FC). The optimal threshold for the L2FC cutoff was determined by optimizing the F1-score of the CRISPRi essentiality predictions obtained by varying L2FC thresholds and comparing these against the TnSeq predictions of essentiality. The optimal threshold was estimated at L2FC <−5.1 at 24.3 generations. Genes exceeding both thresholds (i.e., L2FC <−5.1 and $p_{adj}<0.01$) were called as CRISPRi essential genes by our methodology.

#### Estimate of the “bad-seed” effect

Bad-seeds were originally identified by Cui et al. (2018) as a poorly understood sequence-specific toxicity effect determined by the five PAM-proximal bases of the sgRNA targeting sequence. To evaluate whether such an effect was also seen in our experiment, all possible PAM-proximal 5-nucleotide sequences were treated as putative bad-seeds. An approach similar to Cui et al. was taken and a two-tailed t-test was used to compare: (1) the L2FC of sgRNAs targeting genes predicted to be non-essential by TnSeq that contained any of the putative bad-seeds against (2) the average L2FC among all guides at 24.3 generations. 109 out of 1,018 PAM-proximal 5-nucleotide sequences were identified as leading to significant L2FC differences (adjusted p-value < 0.01). Of these 109 putative bad-seeds, only 12 had a L2FC < –1, with most of them (85 of 109) having a positive L2FC. As the top putative bad-seeds identified in our analysis differed from those identified in Cui et al. (2018), we next focused our analysis on the top 5 bad-seeds identified using SpydCas9 in their work. A two-tailed t-test was used to compare the L2FC between sgRNAs containing their bad-seed sequences and sgRNAs without a known bad-seed. To minimize the possibility of confounding effects, only TnSeq non-essential genes that had at least one sgRNA containing one of the bad-seeds were included in the focused analysis.

#### Quantification of synthesis errors

Errors that occurred during oligo array synthesis or library propagation were identified by looking for sequencing reads that had a single mutation within the sgRNA targeting sequence as compared to a designed sgRNA in RLC12. Synthesis errors were associated with their corresponding “matched” sgRNA, and the position of the mismatch within the sgRNA targeting sequence was identified. To determine the effect of the mutation on mismatch sgRNA depletion, the difference between L2FC values (see Quantification of sgRNA depletion) for the sgRNAs containing the synthesis error and the corresponding matched sgRNA was taken (ΔL2FC). This analysis was restricted to matched sgRNAs that depleted from the library (L2FC < –2 by 24.3 generations), since non-depleting sgRNAs are uninformative for this analysis. Furthermore, in an attempt to distinguish Illumina sequencing errors from errors in oligo array synthesis or library propagation, the analysis was limited only to those sgRNAs which had at least 20 counts in the –ATc condition. The mean ΔL2FC was then estimated for synthesis errors occurring at different positions within the sgRNA targeting sequence over the course of the Mtb CRISPRi screen, indicating the average effect of mutations on sgRNA depletion.

#### Vulnerability model

Vulnerability was estimated by applying a Bayesian multilevel model to each gene, implemented in Stan (version 2.19.3) (Carpenter et al., 2017, Stan Development Team, 2020, Stan Development Team, 2021). The model contained two levels: an sgRNA-level and a gene-level. The sgRNA-level consisted of the two-line model described in Modeling sgRNA depletion over time to model depletion of individual sgRNAs targeting the given gene.The gene-level consisted of a logistic curve to describe the relationship of the sgRNA level depletion over all the guides and their predicted strength, ultimately leading to gene-level estimates of vulnerability.

To make the model more robust to outliers, the mean L2FC values, $y_{i}$, from the individual sgRNAs targeting a gene were modeled as coming from the t-distribution:

$$y_{i}\;∼\;student\_t\left(\upsilon_{y},\;\mu_{i}\;,\sigma \right)$$

where $\upsilon_{y}$ is a gene-level parameter governing the degrees-of-freedom of the distribution, μ is a vector of the sgRNA level parameters for each data point, and σ is a gene-level standard deviation for the t-distribution. At a given data-point i, belonging to a specific sgRNA, j, its mean, $\mu_{i}$ is defined as follows (see *Modeling sgRNA depletion over time* for details):

$$\mu_{i}\;=\;\alpha_{l}^{j}\;+\;\beta_{l}^{j}\text{X }\;if\;x_{i}\;\leq \;\gamma$$

$$\mu_{i}\;=\;\left(\alpha_{l}^{j}+\;\beta_{l}^{j}\gamma^{j}\right)+\;\beta_{e}^{j}*\left(x_{i}-\gamma^{j}\right)\;if\;x_{i}\;>\;\gamma$$

Each of these parameters is assigned their own prior distribution. To capture the relationship between the predicted strength of individual sgRNAs (S) and their rate of depletion, we assigned the following hierarchical prior for the $\beta_{e}$ values:

$$\;\beta_{e}∼\;student\_t\left(\nu_{\beta_{e}},\;\mu_{\beta_{e}},\sigma_{\beta_{e}}\right);$$

where $\nu_{\beta_{e}}$ determines the degrees-of-freedom of the t-distribution, $\sigma_{\beta_{e}}$ represents the standard deviation, and the mean, $\mu_{\beta_{e}},$ is described by the following four-parameter logistic or Hill curve:

$$\mu_{\beta_{e}}=K+\frac{\left(\beta_{max}-K\right)}{\left(1+\text{exp}\left(-H\;\left(S-M\right)\right)\right)}$$

where S is a vector of the predicted strength values for all sgRNAs targeting the gene, K represents the upper limit of the logistic curve, $\beta_{max}$ represents the lower limit of the logistic curve (i.e., the strongest depletion $\left(\beta_{e}\right)$ predicted by the logistic curve), M represents the mid-point of the logistic curve (i.e., the predicted strength where the median $\beta_{e}$ is found), and H is the Hill-coefficient, which governs how fast the logistic curve goes from upper to lower limit (see Figure 3A). See source code under Data and code availability for more details.

This hierarchical model was fit using the Variational Inference functionality in Stan, which implements the Automatic Differentiation Variational Inference (ADVI) algorithm (Kucukelbir et al., 2017). ADVI provides approximate Bayesian inference for complex models that are not feasible in traditional MCMC sampling. A total of 5,000 samples was taken estimate the posterior distribution of all parameters.

To estimate a single value that represents the vulnerability of a gene, the L2FC values predicted by our model for hypothetical guides of different strengths were summed to give a measure of depletion as a function of vulnerability. In specific, the following equations were used to estimate the L2FC at 25 generations for theoretical guides of varying strengths:

$$\text{Logistic}\left(s\right)=\;K+\frac{\left(\beta_{max}-K\right)}{\left(1+\text{exp}\left(-H\;\left(S-M\right)\right)\right)}$$

$$\text{TwoLine}\left(\beta_{e}\right)\;=\;\left(\alpha_{l}\;+\;\beta_{l}\gamma \right)+\;\beta_{e}*\left(25-\gamma \right)$$

where Logistic(s) is the logistic curve implied by the parameters estimated by our model and relates a strength, s, to a rate of depletion $\left(\beta_{e}\right)$, and $\text{TwoLine}\left(\beta_{e}\right)$takes a rate of depletion $\left(\beta_{e}\right)$ and predicts the L2FC at 25 generations, given the estimates of the gene-level parameters. Together these two functions can predict the L2FC at 25 generations for guide of a given strength (assuming gene-level estimates of all other parameters). To consider the implied behavior of sgRNAs of all possible strengths (regardless of whether they can be constructed or not), the definite integral of the composite of the two functions was obtained:

$$\overset{1}{\underset{0}{\int }}\text{TwoLine}\left(\text{Logistic}\left(s\right)\right)\;ds$$

For each gene, the infinite sum described by this integral (which has an analytical solution) was used as a measure of vulnerability. Genes were considered to have high-confidence vulnerability estimates if they met the following criteria:

• The span of the HDI for the M parameter must be < 0.2 (i.e., $HDI_{upper}\left(M\right)\;-\;HDI_{lower}\left(M\right)<\;0.2$)
• The span of the HDI for the $\beta_{max}$ parameter must be < 0.2 (i.e., $HDI_{upper}\left(\beta_{max}\right)-\;HDI_{lower}\left(\beta_{max}\right)<\;0.2$)
• The gene must contain guides spanning a range of strengths of at least 0.5 (i.e., max(S)−min(S)>0.5)

#### Evolutionary Analysis

We studied gene evolution both within *M. tuberculosis* and between different bacterial species. For the *M. tuberculosis* species-level evolutionary analysis, we took the gene level dN/dS ratio estimates (geometric mean (ω)) obtained by a model-averaged analysis of 10,209 Mtb clinical strains from reference (Wilson and CRyPTIC Consortium, 2020). We then compared genes predicted to be essential (n = 624) and non-essential (n = 3,201) by TnSeq (DeJesus et al., 2017) using a two-tailed t-test. We likewise compared CRISPRi essential genes with a high-confidence call ranking (n = 543) in the Mtb upper (n = 136) and lower (n = 136) vulnerability quartiles to all TnSeq essential genes.

To study gene conservation across different bacterial species, we ran BLAST (Altschul et al., 1990) searches comparing the annotated protein sequences from the Mtb genome against each of the eight bacterial genomes we chose for comparison (GenBank: NC_002945.3: *M. bovis;* GenBank: NC_011896.1: *M. leprae;* GenBank: NC_008595.1: *M. avium*; GenBank: NC_008596.1: *M. smegmatis;* GenBank: NC_010397.1: *M. abscessus;* GenBank: NC_022040.1: *C. glutamicum*; GenBank: NC_000964.3: *B. subtilis*; and GenBank: NC_000913.3: *E. coli*). *blastp* was run such that it filtered results to those that had e-values < 0.0001 and so that it reported protein similarity (ppos). The average similarity of homologs was obtained by taking the output % positive (“ppos”) column in the tabular output. For each gene, the match with the highest % similarity was taken as the homolog. Next, the average ± SEM % similarity was calculated for genes ranking in the Mtb upper and lower vulnerability quartiles. This approach was used to determine the average similarity of homologs between species. Genes with no homologs were not considered in this species-level calculation. The number of genes with a homolog was listed separately. Lastly, we compared gene essentiality between species. Genome-wide essentiality information was available for M. *tuberculosis* (DeJesus et al., 2017), *M. smegmatis* (Dragset et al., 2019), *C*. *glutamicum* (Lim et al., 2019), *B. subtilis* and *E. coli* (Koo et al., 2017). We quantified the number of essential Mtb genes ranking in the upper and lower quartile of vulnerability with an essential homolog in any of the other species.

#### Pathway Analysis

Pathway enrichment was restricted to *M. tuberculosis* and *M. smegmatis* genes for which high confidence vulnerability assessments (see Vulnerability model) could be made. First, all annotated genes were associated with a Subsystem subclass as defined by the Pathosystems Resource Integration Center (PATRIC) (Davis et al., 2020). Where necessary, annotations were hand-curated to update the functional categories for genes that were misannotated or not annotated at all. A final total of 455 *M. tuberculosis* genes and 316 *M. smegmatis* genes had a functional annotation. Next, genes were divided genes into two groups: the top quartile most vulnerable and bottom quartile least vulnerable gene sets (n = 114 for Mtb; n = 79 for Msmeg). Enrichment of the categories represented in each group was calculated by an odds ratio and significance was determined with a Fisher’s exact test. Only categories with 5 or more genes and odds ratio > 3 were retained.

### Additional resources

The vulnerability predictions for all targeted *M. tuberculosis* and *M. smegmatis* genes and associated visualization plots are available at https://pebble.rockefeller.edu.

## Supplemental information

Table S1. Plasmids, sgRNA targeting sequences, deep sequencing primers, and qPCR primers used in this work, related to Figures 1, 2, 3, 4, 6, S1, S3, and S6

Data S1. sgRNA two-line parameter fits and linear model coefficients for Mtb H37Rv, HN878, and Msmeg, related to Figures 2, 4, S1, and S6

Data S2. Essentiality calls and VI values for all targeted Mtb H37Rv, HN878, and Msmeg genes, related to Figures 1, 3, 4, 5, 6, S2, S3, S4, S5, and S6
